# An update of the Worldwide Integrated Assessment (WIA) on systemic insecticides. Part 1: new molecules, metabolism, fate, and transport

**DOI:** 10.1007/s11356-017-0394-3

**Published:** 2017-11-05

**Authors:** Chiara Giorio, Anton Safer, Francisco Sánchez-Bayo, Andrea Tapparo, Andrea Lentola, Vincenzo Girolami, Maarten Bijleveld van Lexmond, Jean-Marc Bonmatin

**Affiliations:** 1 Laboratoire Chimie de l’Environnement, Centre National de la Recherche Scientifique (CNRS) and Aix Marseille University, Marseille, France; 2 Institute of Public Health, Ruprecht-Karls-University, INF324, 69120 Heidelberg, Germany; 3 School of Life and Environmental Sciences, The University of Sydney, 1 Central Avenue, Eveleigh, NSW 2015 Australia; 4 Dipartimento di Scienze Chimiche, Università degli Studi di Padova, 35131 Padua, Italy; 5 Task Force on Systemic Pesticides (TFSP), 46 Pertuis-du-Sault, 2000 Neuchâtel, Switzerland; 6 Centre de Biophysique Moléculaire, Centre National de la Recherche Scientifique (CNRS), Rue Charles Sadron, 45071 Orléans, France

**Keywords:** Systemic insecticides, Neonicotinoids, Fipronil, Mode of action, Metabolites, Synergy, Residues, Remediation, Review

## Abstract

With the exponential number of published data on neonicotinoids and fipronil during the last decade, an updated review of literature has been conducted in three parts. The present part focuses on gaps of knowledge that have been addressed after publication of the Worldwide Integrated Assessment (WIA) on systemic insecticides in 2015. More specifically, new data on the mode of action and metabolism of neonicotinoids and fipronil, and their toxicity to invertebrates and vertebrates, were obtained. We included the newly detected synergistic effects and/or interactions of these systemic insecticides with other insecticides, fungicides, herbicides, adjuvants, honeybee viruses, and parasites of honeybees. New studies have also investigated the contamination of all environmental compartments (air and dust, soil, water, sediments, and plants) as well as bees and apicultural products, food and beverages, and the exposure of invertebrates and vertebrates to such contaminants. Finally, we review new publications on remediation of neonicotinoids and fipronil, especially in water systems. Conclusions of the previous WIA in 2015 are reinforced; neonicotinoids and fipronil represent a major threat worldwide for biodiversity, ecosystems, and all the services the latter provide.

## Introduction

In January 2015, a comprehensive set of papers on the environmental impacts of neonicotinoids and fipronil was published (Bijleveld van Lexmond et al. 2015). Since then, the amount of research papers concerning these systemic insecticides has been growing fast. Hundreds of scientific papers dealing with environmental issues of neonicotinoids and fipronil are published every year. This calls for an update of the previous review, which is now presented in three papers in this journal volume.

The first review paper deals with the mode of action of neonicotinoids and fipronil, their metabolism, synergies with other pesticides, degradation products and their contamination of the environment, including new insecticides launched to the market that had not been covered in the previous review. The second paper covers their effects on organisms, from aquatic and terrestrial invertebrates to vertebrates, and their impacts on ecosystems (Pisa et al. 2017). The third paper discusses the efficacy of neonicotinoids and fipronil in agriculture and proposes some alternatives to pest control (Furlan et al. 2017).

## Molecules

**Fig. 1:**
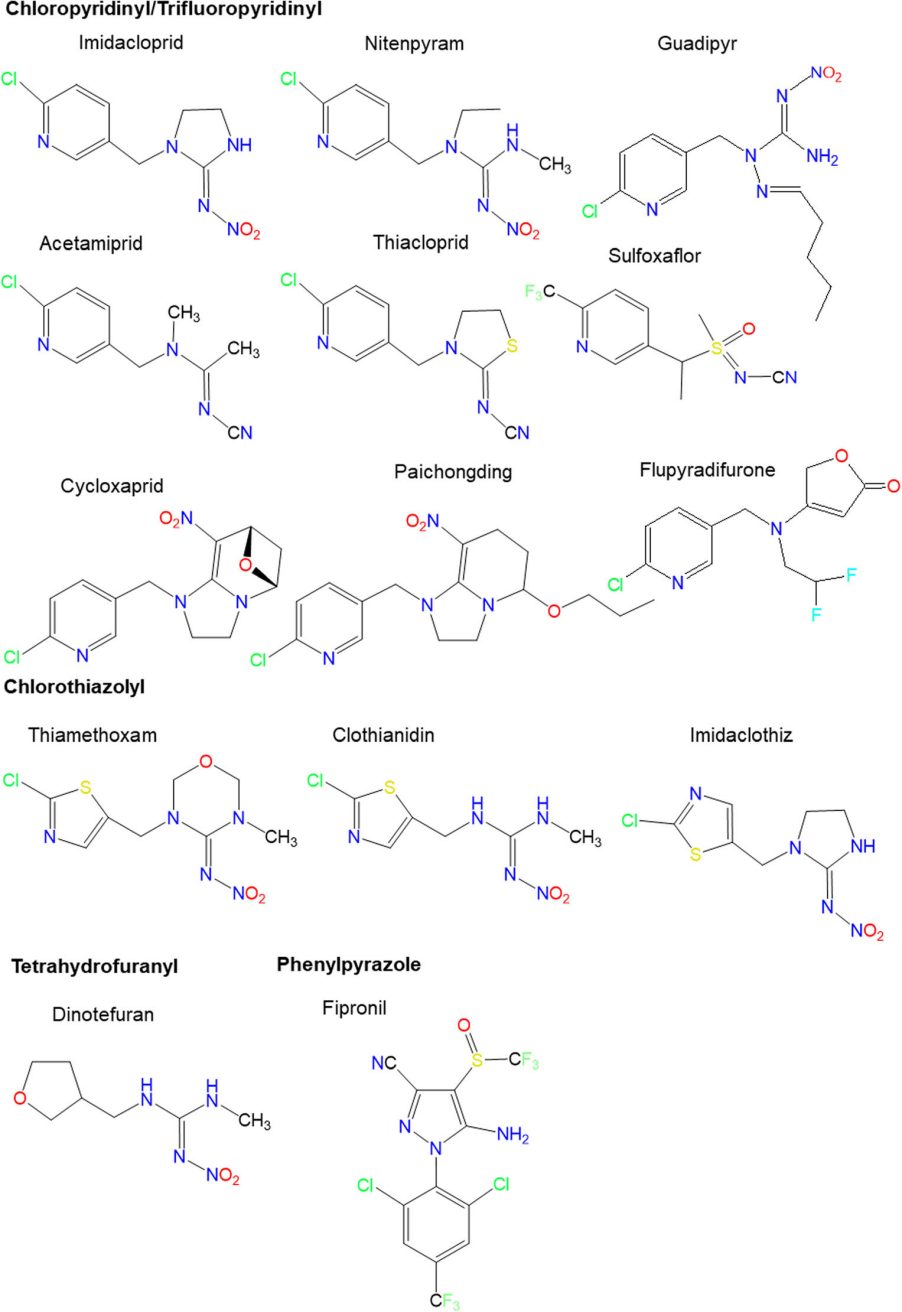
Common names and molecular structures of neonicotinoids and fipronil, depicted by functional groups. Updated from Simon-Delso et al. (2015)

The current paper is focused on the neonicotinoid compounds imidacloprid, clothianidin, thiamethoxam, nitenpyram, acetamiprid, thiacloprid, and dinotefuran, and the phenyl-pyrazole fipronil considered in the initial WIA paper (Simon-Delso et al. 2015). Additionally, the newly marketed fourth-generation neonicotinoid compounds cycloxaprid, imidaclothiz, paichongding and sulfoxaflor, guadipyr, and flupyradifurone have been included. Molecular structures of the insecticides covered in this review are shown in Fig. 1.

Recent advances in regard to the mode of action and metabolism of all these compounds in invertebrates and vertebrates are reviewed here. A broad-scaled literature search was performed using the Web of Science™ and Scopus®. Search terms were [product] and “mode of action,” “metabolism,” “neonicotinoid,” “synergy,” and “metabolite,” where [product] was a placeholder for the name of each considered active ingredient (a.i.).

### Mode of action and metabolism

Neonicotinoids act as agonists on nicotinic acetylcholine receptors (nAChRs). Differences in properties and structures of the receptors in arthropods and mammals explain at least partly the differential selectivity and toxicity toward various taxa (Simon-Delso et al. 2015; Tomizawa et al. 2000). The regions of nAChRs involved in the binding to the α and β subunits are named loops (A, B, C, D, E, and F) and constitute the “binding pocket” (Guo et al. 2015; Ihara et al. 2014; Ihara et al. 2015). Fipronil instead acts as antagonist of the GABA receptors and glutamate-gated chloride channels. Glutamate-gated channels are specific to invertebrates, which explains why fipronil is more toxic to invertebrates than vertebrates (Simon-Delso et al. 2015). Flupyradifurone is a newly developed systemic insecticide (Jeschke et al. 2015). Despite being proposed by the manufacturer as a “butenolide insecticide” rather than a neonicotinoid, the flupyradifurone mode of action is comparable to that of the neonicotinoids, i.e., agonistic binding to insect nAChRs. Flupyradifurone has a chemical structure partially overlapping with the neonicotinoids imidacloprid, nitenpyram, acetamiprid, and thiacloprid (Nauen et al. 2015; O’Mullane et al. 2015). Metabolites of flupyradifurone include the 6-chloronicotinic acid (6-CNA), common to most neonicotinoids. A very similar situation is observed for sulfoxaflor, introduced as a sulfoximine insecticide by the manufacturer (Sparks et al. 2013). As can be seen in Fig. 1, sulfoxaflor is characterized by functional groups which are in common to, or partially overlapping with, other neonicotinoids. The mode of action is also similar to other neonicotinoids by acting as agonist of nAChRs.

Simon-Delso et al. (2015) reviewed the metabolic pathways of neonicotinoids and fipronil, describing mainly two phases: (i) degradation of the active substance, largely dependent on cytochrome P450; and (ii) formation of conjugates. Here, we report additional data published after submission of the aforementioned WIA review: from 2014 onwards.

#### Invertebrates

We have now a better knowledge on the mode of action of imidacloprid. Previously, it had been assumed that imidacloprid coordinates with the tyrosine residue in loop C of nAChRs, so its guanidine moiety and consecutively the NO_2_ group would form a hydrogen bond with the glutamine residue in loop D. Recently, Ihara et al. (2014) found that neonicotinoids interact additionally with the basic residue of lysine in loop G. The authors predict that neonicotinoid resistance of pests may develop from a mutation from lysine to serine in loop G (Ihara et al. 2014, 2015). Taylor-Wells et al. (2015) found that a potential secondary target of imidacloprid is the GABA receptor Rdl in *Anopheles gambiae*, where imidacloprid acts as an antagonist. Their conclusion is consistent with previous observations that imidacloprid lessens GABA-induced responses in cultured honeybee (*Apis mellifera*) Kenyon cells (Deglise et al. 2002; Taylor-Wells et al. 2015). In addition, imidacloprid decreases the density of the synaptic units in the region of the calyces of mushroom bodies in honeybee brain (Peng and Yang 2016). This finding not only links a decrease in olfactory learning ability to abnormal neural connectivity but also provides evidence that imidacloprid damages the development of the nervous system in regions responsible for both olfaction and vision during the larval stage of the honeybee (Peng and Yang 2016).

It has also been found that both fipronil and imidacloprid are inhibitors of mitochondrial respiration and ATP production in honeybees (Nicodemo et al. 2014), while clothianidin causes rapid mitochondrial depolarization in bumblebees (Moffat et al. 2015), and thiamethoxam alters the thermoregulation of African honeybees (*Apis mellifera scutellata*) (Tosi et al. 2016).

Christen et al. (2016) observed that clothianidin, imidacloprid, thiamethoxam, and acetamiprid led to expressional changes of immune system-related genes in honeybees at environmental realistic concentrations. The investigations covered the single compounds as well as their binary mixtures (Christen et al. 2016). Alterations were found in the brain of experimentally exposed honeybees after exposure up to 72 h. The transcriptional changes of nAChR subunits were identified as upregulation of vitellogenin and downregulation of apidaecin, creb, and pka. The authors suggested that these molecular effects may represent a molecular basis for physiological and behavioral effects such as altered foraging activity (vitellogenin), decreased long-term memory formation (creb and pka), and negative effects on the immune system (apidaecin). Effects were stronger for the three most toxic neonicotinoids to bees, clothianidin, imidacloprid, and thiamethoxam, than for acetamiprid, which can be considered less toxic to honeybees than the others based on the acute LD_50_. The in vivo effects of mixtures exceed agonistic interaction with nAChRs alone, so they are assumed to be a result of interactions with other pathways as well.

Carboxylesterase (CarE) and glutathione S-transferase (GST) are involved in xenobiotic metabolisms in living organisms as detoxification enzymes. Their activity in earthworm *Eisenia fetida* varied with exposure to thiacloprid, being inhibited during exposure and increased over a recovery period in clean soil (Feng et al. 2015). Superoxide dismutase (SOD), catalase (CAT), and peroxidase (POD) are responsible for quenching oxidative stress. Activities of these three enzymes were also inhibited during exposure to imidacloprid (Zhang et al. 2014) and attributed to the accumulation of reactive oxygen species in the tissues. This is likely to be the cause of the DNA damage observed upon exposure to thiacloprid. It was also suggested that long recovery times are needed to return to normal metabolism after thiacloprid intoxication and that the tendency for neonicotinoids to persist in soils reduces the likelihood of this recovery (Feng et al. 2015; Pisa et al. 2015). Accumulation of reactive oxygen species, causing oxidative stress and DNA damage has been observed also for fipronil in in vitro tests on cell lines and was proposed to explain the toxic, mutagenic, recombinogenic and carcinogenic effects of fipronil in *Drosophila melanogaster* before and after metabolization by cytochrome P450 (de Morais et al. 2016). No DNA damage and lower toxicity was associated with guadipyr, a fourth-generation neonicotinoid (Wang et al. 2015a). These authors also tested five neonicotinoids (imidacloprid, acetamiprid, nitenpyram, clothianidin, and thiacloprid) on cellulase activity (enzyme involved in the ability to decompose plant litter and other cellulosic material) in *E. fetida*. They demonstrated that the five neonicotinoids significantly inhibited cellulase activity of the earthworm and they also damaged the epidermal and midgut cells linked to increased mucus and cytothesis, with the strongest effect caused by clothianidin (Wang et al. 2015b).

Vehovszky et al. (2015) tested the pesticide formulations Mospilan (a.i. acetamiprid), Kohinor (a.i. imidacloprid), Actara (a.i. thiamethoxam), and Calypso (a.i. thiacloprid) on cholinergic synapses that exist between the VD4 and RPeD1 neurons in the central nervous system of the pond snail *Lymnaea stagnalis*. They observed that neither of these formulations acted as an acetylcholine (ACh) agonist but showed antagonist activity, inhibiting the cholinergic excitatory components of the VD4-RPeD1 connection (Vehovszky et al. 2015). Exposure to imidacloprid produced perturbations of many biological pathways detected from changes in amino acid and nucleotide metabolites in *L. stagnalis* (Tufi et al. 2015), suggesting that its action is complex and not yet fully understood.

Nonneuronal acetylcholine plays a major role in the reproductive system of mammals (sperm, granulosa cells, placenta, amniotic fluid) as external component of the nonneuronal cholinergic system. Very few facts are known from insects, mostly from bees. The royal jelly produced by the hypopharingeal gland of nursing bees to feed the queen and larvae contains ACh concentrations between 4 and 8 nM. An acidic pH of 4 protects ACh from degradation, while raising the pH to 5.5 lowers the concentration of ACh significantly. Wessler et al. (2016) investigated the effect of 4-week exposure of honeybee (*Apis mellifera carnica*) colonies to high concentrations of clothianidin (100 ng/g or ppb) and thiacloprid (8800 ppb). The result was an 80% decline of ACh release from hypopharingeal glands and in brood food, severely compromising the brood. A second experiment with field-relevant low concentrations of thiacloprid (200 ppb) and clothianidin (1 ppb, 10 ppb) decreased ACh levels in brood food and showed adverse effects in brood development (Wessler et al. 2016).

Chaimanee et al. (2016) used qPCR analysis to quantify expression of genes involved in development, immune responses, and detoxification in honeybee queens and workers 1 day after exposure to imidacloprid and coumaphos. The expression levels of P450 subfamily genes, CYP306A1, CYP4G11, and CYP6AS14, were decreased in honeybee queens treated with coumaphos (5 ppm) and low doses of imidacloprid (20 ppb). Both treatments suppressed the expression of genes related to antioxidation, immunity, and development in queens. Upregulation of antioxidants by these compounds in worker bees was observed at day 1. Coumaphos also caused a repression of CYP306A1 and CYP4G11 in workers. In addition, a sublethal dose of imidacloprid (200 ppb) decreased sperm viability by 50% 7 days after treatment (Chaimanee et al. 2016).

In a field study with three apiaries (63 colonies), designed to investigate the ability of 28 biomarkers as predictors of overwintering strength, imidacloprid (50, 200, 1000 μg/L) significantly reduced the activity of the immune-related enzyme phenoloxidase in forager bee extracts (Wegener et al. 2016). Despite the high doses used, this experiment could not identify significant predictors of overwintering strength other than the 10HDA concentration in worker bee heads.

#### Vertebrates

Stivaktakis et al. (2016) showed that imidacloprid has a genotoxic effect on rabbits. They evaluated parameters of genotoxicity and cytotoxicity by measuring binucleated cells with micronuclei (BNMN), micronuclei (MN), and the cytokinesis block proliferation index (CBPI), in lymphocytes of exposed rabbits. Statistically significant differences in the frequencies of BNMN and MN were observed between control and exposed groups, but there was no dose dependency or time dependency of the genotoxic effect for the administered doses (Stivaktakis et al. 2016).

Neonicotinoids can affect the spatial memory of bats, some of which play an important role as pollinators. Hsiao et al. (2016) tested the impact of imidacloprid on the spatial memory of Formosan leaf-nosed bats, *Hipposideros terasensis*. Six bats were caught in the wild and kept in an experimental chamber sufficiently large (17 × 10 × 5 m) to record flight movements by acoustical tracking. Treated bats received 20 mg/kg dose daily (i.e., ~ 4% of the median lethal dose for rats, 450 mg/kg) and were tested over five consecutive days. Flight paths of echolocation for the treated bats were quite different from their originally learned paths, showing increasing problems in echolocation navigation, whereas the nontreated bats consistently followed an average flight path with little variation. An immune-histochemical analysis showed that neural apoptosis in layers of hippocampal CA1 and MEC areas was significantly increased in treated bats compared with those that received no treatment (*p* < 0.01). Bats treated with imidacloprid could not recover their echolocation ability; moreover, most cells in their hippocampal CA1 and MAC were severely damaged and did not recover (Hsiao et al. 2016).

### Synergy

In natural environments, nontarget species are often exposed to a cocktail of different pesticides in concomitance with other external stressors. Despite the known synergisms of neonicotinoids and fipronil, van der Sluijs et al. (2015) pointed out that a large knowledge gap exists in this regard. New information available is reported below.

#### Additive and enhanced synergistic effects

Sgolastra et al. (2017a) explored the synergistic mortality between clothianidin and nonlethal doses of a fungicide (propiconazole) in three bee species (*Apis mellifera*, *Bombus terrestris*, *Osmia bicornis*) following oral exposure in the laboratory. They found significant synergistic mortality in all three bee species exposed to propiconazole and their respective LD_10_ of clothianidin, with synergistic effects persisting longer in *Osmia bicornis*, the most sensitive species to clothianidin (Sgolastra et al. 2017a). Using commercial formulations of imidacloprid with several pesticides, Zhu et al. (2017) found that mortality to honeybees was increased in mixtures of Advise (58.6 mg a.i./L imidacloprid) + Domark (512.5 mg a.i./L tetraconazole), Advise + Transform (58.5 mg a.i./L sulfoxaflor), and Advise + Vydate (68 mg a.i./L oxamyl), by 20, 15, and 26% respectively. Conversely, mixtures of Advise + Bracket (88.3 mg a.i./L acephate) and Advise + Karate (62.2 mg a.i./L L-cyhalothrin) showed additive interaction, while Advise + Belay (9.4 mg a.i./L clothianidin) and Advise + Roundup (1217.5 mg a.i./L glyphosate) had effects less than additive. The mixture of all eight pesticides sprayed over the worker bees held in cages increased mortality up to 100% and exceeded the additive toxicity by 6% (Zhu et al. 2017).

In fish, Qureshi et al. (2016) exposed groups of aquaria acclimated common carp (*Cyprinus carpio*) to sublethal doses of fipronil and buprofezin (insect growth regulator), singly or in combination (Qureshi et al. 2016). They demonstrated that fipronil and buprofezin insecticides exceed the additive toxicity to the fish when in combination.

Bhaskar and Mohanty (2014) discovered that imidacloprid binds with thyroid hormone receptors in mice, explaining the pesticide-induced hypothyroidism and hyperprolactinemia, and alteration of lipid profile in mice is due to co-exposure of the thyroid-disrupting fungicide mancozeb with imidacloprid. The authors suggest that individual low-dose pesticide exposure might not exert the threshold response to affect the receptors signaling high enough to cause hormonal/metabolic impairment (Bhaskar and Mohanty 2014).

Usaj et al. (2014) measured the growth rates of single deletion mutants of the yeast *Saccharomyces cerevisiae* in the presence of imidacloprid, acetamiprid, or thiacloprid as well as their formulations Confidor, Mospilan, and Actara. They observed that neonicotinoid active substances have a common negative impact on the cell wall organization and biogenesis in yeast and, in most cases, formulations exert more pronounced effects than active substances themselves (Usaj et al. 2014).

Synergism helps decrease the use of active ingredients while maintaining the same level of activity against pests. This is the case for IPPA08, an eight-membered homolog of the cis-neonicotinoid cycloxaprid that can be used as neonicotinoid-specific synergist (Bao et al. 2016). However, IPPA08 increases the toxicity of several neonicotinoid insecticides (i.e., acetamiprid, thiacloprid, clothianidin, and imidacloprid) to nontarget species as well, such as honeybees and the brown planthopper (*Nilaparvata lugens*) (Bao et al. 2016). Also, the root fertilizer “Root Feed” (i.e., 9% N, 7% Ca, 1.5% Mg, and 0.1% B) subirrigated in the growing medium has been able to enhance imidacloprid efficacy against the whitefly on tomato (Sun and Liu 2016).

#### Interactions with other stressors

In addition to the above synergies, neonicotinoids and fipronil interact with or promote natural stressors, too. Di Prisco et al. (2016) found that *deformed wing virus* (DWV) adversely affects humoral and cellular immune responses in honeybees. This immunosuppressive effect of the viral pathogen enhances reproduction of the parasitic mite, triggering a loop interaction with escalating negative effects. Chaimanee et al. (2016) also found that DWV replication is increased in worker bees that have been treated with imidacloprid, so exposure to neonicotinoids may exacerbate this mechanism and synergistically contribute to the colony collapse. In a recent review, Sánchez-Bayo et al. (2016b) have highlighted that immune suppression of the natural defenses in bees by neonicotinoid and fipronil insecticides opens the way to parasite infections and viral diseases. Thus, exposure to these pesticides is a key factor contributing to the increasing negative impact of parasitic infections observed in bees throughout recent decades (Aufauvre et al. 2014). In other words, it is very likely that exposure to neonicotinoids and fipronil can boost pathogenicity of some natural infectious agents which otherwise would remain asymptomatic (Goulson et al. 2015; ANSES 2015).

Imidacloprid and *Varroa* mite interactions were investigated by Alburaki et al. (2015). They found higher pathogen and *Varroa* mite loads in hives near corn crops treated with neonicotinoids. The same interaction was also investigated by Abbo et al. (2017). The study provides clear evidence that the triangle of *Varroa destructor*, DWV, and imidacloprid interact and can result in disastrous health and survival effects in honeybees. The mite is the vector for the DWV infection. Sublethal exposure to the neonicotinoid enhances the virus replication (Di Prisco et al. 2013) and also might lead to increased energy stress for detoxification (Abbo et al. 2017). This study shows a significant reduction of vitellogenin (Vg) titer in honeybees that have been exposed to imidacloprid, and Vg is linked to energy homeostasis. In this context, it should be mentioned that Nicodemo et al. (2014) showed that fipronil and imidacloprid impair energy production in mitochondria.

Dussaubat et al. (2016) studied sublethal effects of imidacloprid together with the widely distributed microsporidian parasite *Nosema ceranae* on queen’s physiology and survivorship, both under laboratory and field conditions. The study showed that combined neonicotinoid pesticide and parasite stress alter honeybee queens’ physiology and survival. Doublet et al. (2014) experimentally assessed the interactions between two common microbiological pathogens and thiacloprid in honeybee colonies by a full-factorial design. They found that adult worker bee mortality is increased by two synergistic interactions: one between *Nosema ceranae* and *black queen cell virus* (BQCV) and another between thiacloprid and *Nosema ceranae* (Doublet et al. 2014). The thiacloprid-*Nosema* interaction impaired larval survival, likely because the pesticide elevated viral loads significantly.

#### Antagonistic effect

In some cases, combinations of pesticides may exert an adverse effect that is less than additive. Concerning neonicotinoids, only a couple of examples showed this and only for very specific mechanisms. Bianchi et al. (2015) tested the individual and combined genotoxic potential of imidacloprid and the herbicide sulfentrazone on hepatoma cells lines (HepG2). While the individual pesticides caused irreparable alterations in the cells, the combination of the two pesticides showed an antagonistic effect in the comet assay, and the damage induced was milder and not persistent. The fluorescence in situ hybridization method in HepG2 cells revealed that the damage measured in the micronucleus test resulted from clastogenic effects of imidacloprid.

Christen et al. (2017) tested the effect of binary mixtures of the neonicotinoids acetamiprid, clothianidin, imidacloprid, and thiamethoxam on transcriptional induction of nAChRs in honeybees and found that binary mixtures did not show additive transcriptional inductions but were instead less than additive (Christen et al. 2017). However, in vivo effects are not only governed by agonistic interaction with nAChRs but including complex interactions with other pathways and stressors.

In the case of mixtures of commercial formulations, the lethal effects of Advise (58.6 mg a.i./L imidacloprid) + Belay (9.4 mg a.i./L clothianidin) and Advise + Roundup (1217.5 mg a.i./L glyphosate) were less than additive (Zhu et al. 2017).

### Metabolites, degradation products, and pathways

Degradation products and metabolites of neonicotinoid insecticides and fipronil have been exhaustively described in a previous review (Simon-Delso et al. 2015). Additional new data included here concern mainly cis-neonicotinoids and fourth-generation neonicotinoids, while no extensive additional literature has since been published on newly discovered degradation products and metabolites of imidacloprid, nitenpyram, clothianidin, thiamethoxam, dinotefuran, acetamiprid, thiacloprid, and fipronil. Previously unreported metabolites are listed in Table 1.

**Table 1:** New metabolites of neonicotinoids and fipronil in addition to those reported by Simon-Delso et al. (2015)

Parent compound	Metabolites	Formation medium	References
Imidacloprid	Carbonyl derivative, N-{1-[(6-chloropyridin-3-yl)methyl]-(4 or 5)-oxoimidazolidin-2-ylidene}nitramide	*D. melanogaster*	Hoi et al. (2014)
	Hydroxy desnitro imidacloprid, (1 or 3)-[(6-chloropyridin-3-yl)methyl]-2-iminoimidazolidin-4-ol	*D. melanogaster*	Hoi et al. (2014)
Nitenpyram	Deg_01, N-[(6-chloropyridin-3-yl)methyl]ethanamine	Drinking water	Noestheden et al. (2016)
	Deg_03, N-[(6-chloropyridin-3-yl)methyl]-N-ethyl-N′-methylmethanimidamide	Drinking water	Noestheden et al. (2016)
	Deg_04, N-[(6-chloropyridin-3-yl)methyl]-N-ethyl-N′-methylurea	Drinking water	Noestheden et al. (2016)
	Deg_14, (E)-N1-[((4 or 5),6-dichloropyridin-3-yl)methyl]-N1-ethyl-N′1-methyl-2-nitroethene-1,1-diamine	Drinking water	Noestheden et al. (2016)
	Deg_16, (E)-N′1-[(2-chloropyridin-4-yl)methyl]-N1-[(6-chloropyridin-3-yl)methyl]-N1-ethyl-2-nitroethene-1,1-diamine	Drinking water	Noestheden et al. (2016)
	Deg_18, (1E)-N′1-[(2-chloropyridin-4-yl)methyl]-N1-[(6-chloropyridin-3-yl)methyl]-N1-ethyl-2-nitrobut-1-ene-1,1-diamine	Drinking water	Noestheden et al. (2016)
Fipronil	M7, 1-[2,6-dichloro-4-(trifluoromethyl)phenyl]-5-imino-4-oxo-4,5-dihydro-1H-pyrazole-3-carbonitrile	Rat urine	McMahen et al. (2015)
	M4, 1-[2,6-dichloro-4-(trifluoromethyl)phenyl]-5-nitroso-1H-pyrazol-4-ol	Rat urine	McMahen et al. (2015)
	Hydroxylated fipronil sulfone, 5-amino-1-[2,6-dichloro-3-hydroxy-4-(trifluoromethyl)phenyl]-4-[(trifluoromethyl)sulfonyl]-1H–pyrazole-3-carbonitrile	Fungal degradation (batch reactor)	Wolfand et al. (2016)
	Glycosylated fipronil sulfone, 5-amino-1-[2,6-dichloro-3-{[3,4-dihydroxy-5-(hydroxymethyl)tetrahydrofuran-2-yl]oxy}-4-(trifluoromethyl)phenyl]-4-[(trifluoromethyl)sulfonyl]-1H–pyrazole-3-carbonitrile	Fungal degradation (batch reactor)	Wolfand et al. (2016)
	TP3, {(E)-[2,6-dichloro-4-(trifluoromethyl)phenyl]diazenyl}acetonitrile	Photocatalytic degradation in water	Gomes Júnior et al. (2017)
	TP4, 5-amino-4-[(trifluoromethyl)sulfonyl]-1H–pyrazole-3-carbonitrile	Photocatalytic degradation in water	Gomes Júnior et al. (2017)
Cycloxaprid	TP1, 9-nitro-1-[(pyridin-3-yl)methyl]-2,3,5,6-tetrahydro-1H-imidazo[1,2-a]azepine-(2 or 3),5-diol	Soil	Liu et al. (2015)
	TP2, 2,3-dihydroxy-9-nitro-2,3,6,7-tetrahydro-1H-imidazo[1,2-a]azepine-5,8-dione	Soil	Liu et al. (2015)
	TP3, 2,3,9-trihydroxyhexahydro-1H-imidazo[1,2-a]azepine-5,8-dione	Soil	Liu et al. (2015)
	TP4, 1-{1-[(6-chloropyridin-3-yl)methyl]-(4 or 5)-hydroxyimidazolidin-2-yl}-3-hydroxypropan-1-one	Soil	Liu et al. (2015)
	TP5, 1-[(6-chloropyridin-3-yl)methyl]imidazolidine-2-carbaldehyde	Soil	Liu et al. (2015)
	TP6, 1-[(6-chloropyridin-3-yl)methyl]-(4 or 5)-hydroxyimidazolidine-2-carboxylic acid	Soil	Liu et al. (2015)
	TP7, 2-chloro-8-hydroxy-7,8-dihydro-1,6-naphthyridine-6(5H)-carbaldehyde	Soil	Liu et al. (2015)
	TP8, (2Z)-2-(3-hydroxy-1-nitrosopropylidene)-(1 or 3)-[(pyridin-3-yl)methyl]imidazolidin-4-ol	Soil	Liu et al. (2015)
	TP9, (2Z)-2-(2-hydroxy-1-nitrosoethylidene)-(1 or 3)-[(pyridin-3-yl)methyl]imidazolidin-4-ol	Soil	Liu et al. (2015)
	TP10, (2Z)-2-(2-hydroxy-1-nitrosoethylidene)-1-[(pyridin-3-yl)methyl]imidazolidine-4,5-diol	Soil	Liu et al. (2015)
	TP11, 1-[(pyridin-3-yl)methyl]imidazolidine-4,5-diol	Soil	Liu et al. (2015)
	M1, (1-(6-chloronicotinyl)-2- nitromethylene-imidazolidine), (nitromethylene)imidazole, (2Z)-1-[(6-chloropyridin-3-yl)methyl]-2-(nitromethylidene)imidazolidin-4-ol	Aerobic soil	Chen et al. (2017)
	M2, 1-[(6-chloropyridin-3-yl)methyl]-4-hydroxy-4,5-dihydro-1H-imidazole-2-carboxylic acid	Aerobic soil	Chen et al. (2017)
	M3, 1-[(6-chloropyridin-3-yl)methyl]imidazolidine-2,4-diol	Aerobic soil	Chen et al. (2017)
Imidaclothiz	Olefin imidaclothiz, N-{1-[(2-chloro-1,3-thiazol-5-yl)methyl]-1,3-dihydro-2H-imidazol-2-ylidene}nitramide	Soil (microbial)	Liu et al. (2011)
	Nitroso imidaclothiz, N-{1-[(2-chloro-1,3-thiazol-5-yl)methyl]imidazolidin-2-ylidene}nitrous amide	Soil (microbial)	Liu et al. (2011)
	Guanidine imidaclothiz, seco imidaclothiz, N-[(2-chloro-1,3-thiazol-5-yl)methyl]-N″-nitroguanidine	Soil (microbial)	Liu et al. (2011)
Paichongding	1-((6-Chloropydidin-3-yl)methyl)-7-methyl-8-nitro-5-hydroxy-1,2,3,5,6,7-hexahydroimidazo[1,2-α]pyridine	Soil (microbial)	Cai et al. (2015a, b)
	1-((6-Chloropydidin-3-yl)methyl)-7-methyl-8-hydroxy-5-propoxy-1,2,3,5,6,7-hexahydroimidazo[1,2-α]pyridine	Soil (microbial)	Cai et al. (2015b)
	1-((6-Chloropydidin-3-yl)methyl)-7-methyl-5-carbonyl-1,2,3,5,6,7-hexahydroimidazo[1,2-α]pyridine	Soil (microbial)	Cai et al. (2015b)
	1-((6-Chloropydidin-3-yl)methyl)-7-methyl-8-amino-1,2,3,5,6,7-hexahydroimidazo[1,2-α]pyridine	Soil (microbial)	Cai et al. (2015b)
	8-Amino-1,2,3,5,6,7-hexahydroimidazo[1,2-α]pyridine	Soil (microbial)	Cai et al. (2015b)
	M5, 1-(6-chloropyridin-3-ylmethyl)-7-methyl-8-nitroso-5-propoxy-1,2,3,5,6,7-hexahydroimidazo[1,2-α]pyridine	Soil (microbial)	Cai et al. (2015a)
		Flooded paddy soil	Li et al. (2016a)
	1-(6-Chloropyridin-3-ylmethyl)-7-methyl-5-propoxy-1,2,3,5,6,7-hexahydroimidazo[1,2-α]pyridine	Soil (microbial)	Cai et al. (2015a)
	1-((6-Chloropyridin-3-yl)methyl)-5,7-diol-8-amino-1,2,3,5,6,7-hexahydroimidazo[1,2-α]pyridine	Soil (microbial)	Cai et al. (2015a)
	1-((6-Chloropyridin-3-yl)methyl)-5,7-diol-8-amino-octahydroimidazo[1,2-α]pyridine	Soil (microbial)	Cai et al. (2015a)
	I4, 1-((6-chloropyridin-3-yl)methyl)-2,3-dihydro-5-one-7-methylimidazo[1,2-α]pyridine	Soil (microbial)	Cai et al. (2015a)
		Biodegradation in water in dark conditions	Wang et al. (2016)
	I1, M1, 1-(6-chloropyridin-3-ylmethyl)-7-methyl-8-nitro-1,2,3,5,6,7- hexahydroimidazo[1,2-α]pyridin-5-ol	Biodegradation in water in dark conditions	Wang et al. (2016)
		Flooded paddy soil	Li et al. (2016a)
	I2, 8-amino-1-(6-chloropyridin-3-ylmethyl)-octahydroimidazo[1,2-α]pyridine-5,7-diol	Biodegradation in water in dark conditions	Wang et al. (2016)
	I3, 8-amino-1-(6-chloropyridin-3-ylmethyl)octahydroimidazo[1,2-α]pyridin-7-ol	Biodegradation in water in dark conditions	Wang et al. (2016)
	I5, octahydroimidazo[1,2-α]pyridin-8-ylamine	Biodegradation in water in dark conditions	Wang et al. (2016)
	I6, 8-nitro-5-propoxy-1,2,3,5,6,7-hexahydroimidazo[1,2-α]pyridine	Biodegradation in water in dark conditions	Wang et al. (2016)
	M2, 1-((6-chloropyridin-3-yl)methyl)-7-methyl-5-propoxyoctahydroimidazo[1,2-α] pyridine	Flooded paddy soil	Li et al. (2016a)
	M3, 1-(6-chloropyridin-3-ylmethyl)-6,7-methyl-8-nitro-1,2,3,5,6,7- hexahydroimidazo[1,2-α]pyridin-5-ol	Flooded paddy soil	Li et al. (2016a)
	M4, 5-hydroxy-7-methyl-1-(pyridin-3-ylmethyl)hexahydroimidazo[1,2-α]pyridin-8(5H)-one	Flooded paddy soil	Li et al. (2016a)
	M6, 1-((6-chloropyridin-3-yl)methyl)-2,3-hydroxy-7-methyl-8-nitro-2,3,6,7-tetrahydroimidazo[1,2-α]pyridin-5(1H)-one	Flooded paddy soil	Li et al. (2016a)
	M7, 1-(6-chloropyridin-3-ylmethyl)-7-methyl-2,3,6,7-tetrahydroimidazo[1,2-α] pyridin-5(1H)-one	Flooded paddy soil	Li et al. (2016a)
	M8, 1-(6-chloropyridin-3-ylmethyl)-7-methyl-8-nitro-5-propoxy-1,2,3,5,6,7-hexahydroimidazo[1,2-α]pyridin-2,3-ol	Flooded paddy soil	Li et al. (2016a)
	M9, 1-(6-chloropyridin-3-ylmethyl)-7-methyl-8-nitro-5-propoxy-1,2,3,5,6,7- hexahydroimidazo[1,2-α]pyridin-6-ol	Flooded paddy soil	Li et al. (2016a)
Sulfoxaflor	X11721061, 1-[6-(trifluoromethyl)pyridin-3-yl]ethan-1-ol	Rice fields and straws	Chung et al. (2017)
		Plants and animals	Pfeil et al. (2011)
	X117119474, N-[methyl(oxo){1-[6-(trifluoromethyl)pyridin-3-yl]ethyl}-λ^4^-sulfanylidene]urea	Rice fields and straws	Chung et al. (2017)
		Soil and plants	Pfeil et al. (2011)
	X11596066, 5-ethyl-2-trifluoromethylpyridine	Animals	Pfeil et al. (2011)
	X11579457, 5-[1-(S-methylsulfonimidoyl)ethyl]-2-(trifluoromethyl)pyridine	Soil	Pfeil et al. (2011)
	X11519540, 5-[(1-methylsulfonyl)ethyl]-2-(trifluoromethyl)pyridine	Soil and animals	Pfeil et al. (2011)
Flupyradifurone	Difluoroacetic acid (DFA)	Soil, water, plants and animals	O’Mullane et al. (2015)
	Flupyradifurone-OH, 4-{[(6-chloropyridin-3-yl)methyl](2,2-difluoroethyl)amino}-5-hydroxyfuran-2(5H)-one	Animals	O’Mullane et al. (2015)
	Flupyradifurone-OH-SA, 3-{[(6-chloropyridin-3-yl)methyl](2,2-difluoroethyl)amino}-5-oxo-2,5-dihydrofuran-2-yl hydrogen sulfate	Animals	O’Mullane et al. (2015)
	Hippuric acid, [(6-chloropyridine-3-carbonyl)amino]acetic acid	Animals	O’Mullane et al. (2015)
	Flupyradifurone-des-difluoroethyl, 4-{[(6-chloropyridin-3-yl)methyl]amino}furan-2(5H)-one	Animals	O’Mullane et al. (2015)
	Difluoroethyl-amino-furanone, 4-[(2,2-difluoroethyl)amino]furan-2(5H)-one	Plants and animals	Li et al. (2016b)
			O’Mullane et al. (2015)
	(6-Chloro-3-pyridyl)methanol*	Plants	O’Mullane et al. (2015)
	6-Chloronicotinic acid*	Plants	Li et al. (2016b)
			O’Mullane et al. (2015)
	Amino-furanone, 4-aminofuran-2(5H)-one	Plants	O’Mullane et al. (2015)
	Flupyradifurone-acetic acid, {[(6-chloropyridin-3-yl)methyl](2,2-difluoroethyl)amino}acetic acid	Plants	O’Mullane et al. (2015)

*Metabolite common to other neonicotinoids

Two new metabolites of imidacloprid were detected that overexpress the gene Cyp6g1 (responsible for upregulation of cytochrome P450, key in neonicotinoid metabolization) in *Drosophila melanogaster*: a carbonyl derivative and hydroxy desnitro imidacloprid (Hoi et al. 2014). A study on the photodegradation of imidacloprid on thin solid films unexpectedly observed a release of N_2_O into the gas phase rather than the expected NO_2_ which may indicate a different reaction mechanism compared to photolysis in solution (Aregahegn et al. 2016). Noestheden et al. (2016) studied the degradation of nitenpyram in unpreserved finished drinking water and showed that its degradation is mediated by oxidation, hydrolysis, and reaction with Cl_2_ leading to the discovery of six reaction products (Noestheden et al. 2016).

McMahen et al. (2015) analyzed fipronil metabolites in rat urines. They discovered two new metabolites (Table 1) formed from oxidation and descyano reaction and from dehydration of the hydroxylamine metabolite already observed in rat urine (Cravedi et al. 2013; McMahen et al. 2015). Wolfand et al. (2016) discovered four new fungal transformation products: hydroxylated fipronil sulfone, glycosylated fipronil sulfone, and two unidentified compounds (Table 1). These were likely formed by enzymatic transformation through hydroxylation of the aromatic ring followed by conjugation with sugar moieties. Gomes Júnior et al. (2017) discovered two new transformation products of fipronil from heterogeneous photocatalysis in water (Table 1).

#### New molecules

Stereoselective soil metabolism of cycloxaprid enantiomers was investigated in four different soils under anoxic and flooded conditions (Liu et al. 2015). The main degradation pathways involved cleavage of the oxabridged seven-member ring, dechlorination in the chloropyridinyl moiety, and cleavage of C–N between the chloropyridinylmethyl and imidazalidine ring producing 11 metabolites (Table 1). Stereoselective transformation was not observed. According to the authors, this could be due to (i) differences arising from oxabridged ring, which did not exhibit distinct physicochemical properties and microbial effects; (ii) all metabolites that underwent cleavage on the oxabridge were no longer chiral molecules; and (iii) soil microbial effects, considered to be a key factor for enantioselectivity, were inhibited under anoxic and flooded condition. TP4 metabolite (Table 1) was the most abundant transformation product (Liu et al. 2015, 2016). Degradation pathways of cycloxaprid have also been studied in soil under aerobic conditions, whereby degradation of cycloxaprid occurs via carboxylation of the alkene group (Chen et al. 2017), as well as by hydroxylation of the imidazolidine ring in addition to the pathways already observed by Liu et al. (2015).

Paichongding, having four stereoisomers, displayed diastereoselective specific mineralization in aerobic soil (Fu et al. 2015). Paichongding is degraded via denitration, depropylation, nitrosylation, demethylation, hydroxylation, and enol-keto tautomerism, producing chiral and biologically active products (Li et al. 2016b). Microbial degradation in soil by a *Sphingobacterium* sp. mainly occurs on the tetrahydropyridine ring and produces five metabolites (Table 1) (Cai et al. 2015b). In anaerobic soils, biodegradation of paichongding occurs via nitro reduction and elimination, hydrolysis, demethylation, and ether cleavage reactions, producing six metabolites (Table 1) (Cai et al. 2015a). Wang et al. (2016) also found that biodegradation in water of SR/RS-paichongding mainly occurred on the tetrahydropyridine ring rather than on the chloropyridine ring. However, in the degradation pathway of RR/SS-paichongding, the breaking of the C–N bond between 2-chloro-5-methylpyridine and 8-amino-octahydroimidazo[1,2-α]pyridin-7-ol and cleavage of the chloropyridine ring were detected. Moreover, the degradation products of SR/RS-paichongding were strikingly different from those of RR/SS-paichongding (Wang et al. 2016). The results are likely caused by the different spatial conformation of the isomer.

Imidaclothiz is transformed in unsterilized soils into the olefin, nitroso, or guanidine derivatives following a degradation pathway which is analogous to that of imidacloprid at the nitroguanidine moiety (Liu et al. 2011).

No information is currently available on degradation products and metabolites of guadipyr.

Metabolization of sulfoxaflor in both animals and plants occurs through oxidative cleavage at the methyl(oxo)sulfanylidene cyanamide moiety, and it can proceed through glucuronidation to form conjugates (Pfeil et al. 2011). The two main metabolites 1-[6-(trifluoromethyl)pyridin-3-yl]ethan-1-ol and N-[methyl(oxo){1-[6-(trifluoromethyl)pyridin-3-yl]ethyl}-λ4-sulfanylidene]urea were detected in rice and rice straws (Chung et al. 2017).

Major metabolites of flupyradifurone are difluoroethyl-amino-furanone and 6-chloronicotinic acid (Li et al. 2016c). The latter is a common degradation derivative of imidacloprid, nitenpyram, acetamiprid, and thiacloprid (Simon-Delso et al. 2015), and it is likely produced from degradation of paichongding and cycloxaprid as they also contain a chloropyridine moiety. Metabolization has been observed to occur at the difluoroethylaminofuranone moiety (O’Mullane et al. 2015).

### Summary of findings

The neonicotinoid family has grown to 13 compounds (imidacloprid, clothianidin, thiamethoxam, nitenpyram, acetamiprid, thiacloprid, dinotefuran, cycloxaprid, imidaclothiz, paichongding, sulfoxaflor, guadipyr, and flupyradifurone) including fourth-generation neonicotinoids and new derivatives.

In regard to the mode of action, new research has shown that neonicotinoids also interact with the basic residue of lysine in loop G in nAChRs and that a secondary target of imidacloprid is the GABA receptor. Flupyradifurone has a mode of action analogous to other neonicotinoids by binding to insect nAChRs and its degradation results in the same suite of metabolites.

Neonicotinoids led to expressional changes of immune system-related genes in honeybees. Imidacloprid decreased the density of the synaptic units in the region of the calyces of mushroom bodies in honeybee brain. A similar effect is observed in a bat species as neural apoptosis in layers of hippocampal CA1 and MEC areas, with the consequence of disturbed spatial navigation. Imidacloprid has genotoxic effects in rabbits and binds with thyroid hormone receptors in mice. Fipronil and imidacloprid are also inhibitors of mitochondrial respiration and ATP production in honeybees. This clearly impacts thermoregulation. The effect is also observed with thiamethoxam. Clothianidin causes rapid mitochondrial depolarization in bumblebees.

Combinations of neonicotinoids have antagonistic effects on transcriptional induction of nAChRs, whereas mixtures with other insecticides usually result in additive effects and interactions with fungicides and other stressors show synergistic effects. Imidacloprid and thiacloprid are likely to enhance virus replication. More studies are needed to investigate the effects of neonicotinoids associated with co-exposure to other xenobiotic substances and environmental stressors and between active ingredients and formulation excipients.

Additional enzymes that may be involved in the metabolism of neonicotinoids are CarE and GST. New metabolites and degradation products were discovered especially for fourth-generation neonicotinoids.

## Environmental contamination

The global output of pesticides is estimated as 6 million tons per year (Bernhardt et al. 2017), with a quarter of the insecticides used being neonicotinoids (Jeschke et al. 2011), while the economic value of the pesticide industry, US$29 billion, “is increasing at a rate more than double that of any other global-change factor”, except the pharmaceutical industry (Bernhardt et al. 2017). Enormous quantities of these chemicals are applied to crops worldwide, and yet a large fraction remains in the soil and contaminates the environment.

In the past two and a half years, a tremendous worldwide effort has provided a clearer picture of the environmental contamination by neonicotinoids and fipronil. There is now increasing awareness of their widespread pollution. Contamination is not just affecting the soils of treated fields but also the neighboring fields and urban areas. The following is an account of the research done since 2014 on the fate and transport routes of neonicotinoids and fipronil systemic insecticides. The residue data detailed below are found in Table 2.

**Table 2:** Residues of neonicotinoids and fipronil in environmental samples. Values indicate the range of concentrations (in ng/g or ng/L, depending on the matrix) and the frequency of detection (%)

Matrix	Acetamiprid	Clothianidin	Dinotefuran	Imidacloprid	Guadipyr	Thiacloprid	Thiamethoxam	Fipronil	Reference
Dust (ng/g)									
Maize planting (Italy)		0.4–905*		11.9–2704*			3.0–940*	1.6–115*	Biocca et al. (2017)
Urban dust (California, USA)								1–6188**	Richards et al. (2016)
Maize planting (Canada)							0.05–8.41**		Xue et al. (2015)
Corn fields (Canada)		17.8–42.3					10.2–65.0		Limay-Rios et al. (2016)
Soil and sediment (ng/g d.w.)									
Canola fields (Midwest USA)		4.4–21.4							Xu et al. (2016)
Cocoa plantation (Ghana)		9.8–23.1 (10%)		4.3–251 (54%)					Dankyi et al. (2014)
Corn field (Midwest USA)		2.0–11.2							de Perre et al. (2015)
Corn fields (Canada)		0.16–0.2					4 ± 1.1		Schaafsma et al. (2015)
Corn fields (Midwest USA)		6.4–20.3							Xu et al. (2016)
Cotton fields (China)								40–650	Wu et al. (2017)
Maize fields (Canada)		2.9–5.1 (100%)					0.3–1.8 (86%)		Limay-Rios et al. (2016)
Oilseed rape (UK)		5.1–28.6 (100%)		0.7–7.9 (100%)		< 0.01–0.2 (43%)	0.5–9.7 (100%)		Botias et al. (2015)
Rice fields (China)		17–600							Li et al. (2014)
Rice fields (Japan)				50–280				10–90	Boulange et al. (2016)
Rice fields (Japan)			25–28						Yokoyama et al. (2015)
Rice fields (Vietnam)				9					La et al. (2015)
River sediment (China)	162 (62.5%)			141 (87.5%)					Chen et al. (2015)
Several crops (Canada)							5.6 ± 0.9		Schaafsma et al. (2016)
Several crops (Central Europe)							72–98		Hilton et al. (2016)
Wheat field margins (UK)		0.4–19.1 (100%)		< 0.07–6.3 (75%)		< 0.01–0.1 (25%)	< 0.04–0.5 (50%)		Botias et al. (2015)
Water (ng/L)									
Arade river (Portugal)				2.5–8.0 (100%)					Gonzalez-Rey et al. (2015)
Corn fields (Canada)		2.28–43.6 (100%)					1.12–16.5 (98%)		Schaafsma et al. (2015)
Drinking water (Iowa, USA)		3.9–57.3 (100%)		1.12–39.5 (100%)			0.2–4.1 (100%)		Klarich et al. (2017)
Ebro river (Spain)				1.1–15.0 (45%)					Ccanccapa et al. (2016)
Forest streams (N Carolina, USA)				29–379 (70%)					Benton et al. (2016)
Groundwater (Wisconsin, USA)		210–3340 (20%)		260–3340 (24%)			200–8930 (55%)		Huseth and Groves (2014)
Infiltration water (Midwest USA)		10–203							de Perre et al. (2015)
Llobregat river (Spain)				2.1–66.5 (78%)					Masiá et al. (2015)
Mekong river (Vietnam)							630–950 (4%)	170–410 (83%)	Chau et al. (2015)
Pothole wetlands (Canada)		310–3500 (98%)		40–120 (48%)			290–6900 (54%)		Evelsizer and Skopec (2016)
Reservoir (Brazil)				< 0.7–3.0 (31%)				1.1–2.0 (91%)	López-Doval et al. (2017)
Rice fields (China)		9.6–166							Li et al. (2014)
Rice fields (China)					0.1–780				Liu et al. (2014)
Rice fields (Japan)			290,000–720,000						Yokoyama et al. (2015)
Rice fields (Japan)				5.0–30				1.3–2.5	Boulange et al. (2016)
Rice fields (Vietnam)				53–83					La et al. (2015)
River (Japan)			10,000						Yokoyama et al. (2015)
Rivers (California, USA)								30–13,800 (100%)	Sengupta et al. (2014)
Runoff water (Midwest USA)		<LOD–850							de Perre et al. (2015)
Rural streams (Germany)				2–20 (32%)			20–44 (10%)		Münze et al. (2015)
Rural streams (Iowa, USA)		8.2–257 (75%)		< 2–42.7 (23%)			< 2–185 (47%)		Hladik et al. (2014)
San Francisco Bay (USA)				13.5–1462 (80%)				1.1–27.4 (81%)	Weston et al. (2015)
Soybean crops (Canada)		3.0–40 (100%)					3.0–1090 (100%)		Chrétien et al. (2017)
Stream (Brazil)							1230–1580 (100%)		Rocha et al. (2015)
Streams (Indiana, USA)		6–671 (96%)		2–177 (90%)			15–2568 (98%)		Miles et al. (2017)
Streams (USA)	1–40 (3%)	34–64 (24%)	4–134 (13%)	5.7–143 (37%)			7–190 (21%)	0.1–10 (84%)	Bradley et al. (2017)
Streams (USA)	2.5–45.6 (7.5%)	1.7–62 (56%)	1.6–4.1 (10%)	2.1–65.9 (87%)			5.6–35.9 (44%)		Hladik and Kolpin (2016)
Sugarbeet crops (Switzerland)				1290			2830		Wettstein et al. (2016)
Wetlands (Canada)	0.6–54.4 (1.5%)	59.7–3110 (76%)		7.1–256 (12%)			40.3–1490 (52%)		Main et al. (2015)
WTP effluent (N Carolina, USA)								10–500 (100%)	McMahen et al. (2016)
Plants (ng/g)									
Cotton seedlings								48–646	Wu et al. (2017)
Foliage (oilseed rape)		1.3–8.7 (100%)		< 0.2–3.1 (2%)			< 0.1–2.6 (100%)		Botías et al. (2016)
Guttation fluid (turfgrass)				23–88					Larson et al. (2015)
Guttation fluid (oilseed rape)		10–132					3.2–12.9		Reetz et al. (2016)
Nectar (canola)		0.3–2.4							Xu et al. (2016)
Nectar (clover) mowed		6.2–18		8.4–26					Larson et al. (2015)
Nectar (clover) sprayed		2882–2992		5493–6588					Larson et al. (2015)
Nectar (oilseed rape)		< 0.17–13.2 (31%)				< 0.03–1.2 (54%)	< 0.1–13.3 (54%)		Botias et al. (2015)
Nectar (oilseed rape)		6.7–16							Rundlöf et al. (2015)
Nectar (oilseed rape)		0.7–0.8							Rolke et al. (2016)
Pollen (beans)							0.2 ± 0.3		David et al. (2015)
Pollen (corn)		1.2–5.7							Xu et al. (2016)
Pollen (oilseed rape)		6.6–23							Rundlöf et al. (2015)
Pollen (oilseed rape)		0.5–0.97							Rolke et al. (2016)
Pollen (oilseed rape)		< 0.12–14.5 (90%)				< 0.04–7.3 (86%)	1.0–11.1 (100%)		Botias et al. (2015)
Pollen (oilseed rape)		< 0.72–11 (73%)				< 0.22–78 (100%)	2.4–11 (100%)		David et al. (2016)
Pollen (raspberries)		6.0 ± 5.9				9.4 ± 2.1	23 ± 38		David et al. (2015)
Pollen (strawberries)		8.9 ± 1.3		3.1 ± 5.4		5.9 ± 0.7	1.5 ± 0.3		David et al. (2015)
Pollen (wildflowers)				< 0.36–1.1 (13%)		< 0.07–4 (63%)	< 0.12–21 (50%)		David et al. (2016)
Pollen collected by honeybees	< 0.07 (4%)	< 0.72 (8%)		< 0.36–3.5 (12%)		< 0.07–10 (48%)	< 0.12–1.6 (64%)		David et al. (2016)
Pollen in apiaries—treated maize fields	0.04–4.7 (28%)	0.64–9.37 (22%)	4.5 (3%)			0.25 (3%)	0.07–0.95 (22%)		Long and Krupke (2016)
Pollen in apiaries—nontreated land		4.7 (3%)	4.8–6.3 (10%)	0.9–1.1 (7%)			0.5–1.7 (10%)		Long and Krupke (2016)

*Units: microgram per cubic meter

**Total residues of parent compound and metabolites

### Air and dust

Sowing of coated seeds generates abraded dust particles containing insecticides. Pneumatic planters are widely used and have been identified as a source of dispersion of abraded particles during maize drilling since 2003 (Greatti et al. 2003). Many other field experiments identified sowing with pneumatic drilling machines as an important source of environmental contamination (Krupke et al. 2012; Pochi et al. 2012; Tapparo et al. 2012). The release into the atmosphere of particulate matter containing insecticides causes the contamination of vegetation surrounding the field, with the consequent exposure of nontarget animals to sublethal dose of insecticides (Greatti et al. 2006; Stewart et al. 2014). Furthermore, these abraded particles pose a serious risk to insects (in particular foraging bees and other pollinators) flying across the field during sowing operations (Girolami et al. 2012, 2013; Marzaro et al. 2011). Since then, attention has been paid to the reduction of particulate matter expulsion using modified drilling machines fitted with devices in order to reduce particulate emissions and proper handling of coated seeds (Biocca et al. 2017; Manzone et al. 2015; Manzone and Tamagnone 2016; Pochi et al. 2015a, b). However, the abrasion potential of seeds still has an important ecological impact without clear benefits in terms of crop yields (Sgolastra et al. 2017b; Zwertvaegher et al. 2016).

Since 2015, several studies have characterized the dust cloud produced from coated seeds. A wide characterization of dust physical-chemical proprieties was done by Foqué et al. (2017a, b). In addition, particulate matter 3D shape has been characterized by means of X-ray micro-CT (Devarrewaere et al. 2015). Information on envelope density, size distribution, and porosity allowed the development of a computational fluid dynamic (CFD) model, which was validated in wind tunnel trials (Devarrewaere et al. 2016). This may help improve our understanding of the atmospheric transport of dust produced during sowing in field-realistic conditions, although actual field trials of exposure may be more convincing than sheer modeling to understand the patterns of bee exposure to such dust (Biocca et al. 2015; Pistorius et al. 2015).

Regarding the environmental contamination due to transport of dusts, residues of thiamethoxam and clothianidin in dust particles comprise some 0.01–0.4% of their actual application rate, with 92% originating from the treated seeds. The neonicotinoid concentration measured in the dust plume is 0.1 μg/m^3^ (Xue et al. 2015). New evidence has shown that pollen and nectar from wild vegetation grown near seed-treated crops are contaminated with highly variable amounts of neonicotinoids, with the consequence of longer exposure for pollinator insects (Botias et al. 2015, 2016; Long and Krupke 2016; Mogren and Lundgren 2016). It is not clear if wild plant contamination is due to atmospheric transport of dusts, soil leaching, or a combination of these factors. However, an accurate analysis of soil and water residues close to maize fields addresses atmospheric transport of dusts as one of the main neonicotinoid sources (Schaafsma et al. 2015). In Ontario corn fields treated with thiamethoxam and/or clothianidin over several years, the mean concentration of neonicotinoids in surface dust before planting was 12.7- to 15.6-fold higher than that in parent soils during two consecutive years: mean concentrations for parent soil beneath and surface dust were 4.36 and 59.86 ng/g (ppb), respectively (Limay-Rios et al. 2016).

Contamination of soil and pavement can also happen through the deposition of atmospheric dust particles and adsorption of volatile fumes (Jiang and Gan 2016). In urban environments, fine dust particles on paved surfaces may be an important source of surface water contamination during wet periods. In California, most dust particles on the driveways, curb gutters, and streets contained pyrethroids (53.5–94.8%) and fipronil (50.6–75.5%) at concentrations in the range of 20–132 ng/g. Concentrations increased with decreasing particle size. This may be due to concentration of residues on the smaller fine particles as they present a bigger surface area and a higher organic carbon content. While the former insecticides are removed by rainfall, fipronil appears to be transformed to its biologically active intermediates on the pavement (Richards et al. 2016).

The atmospheric half-life of fipronil in airborne dust particles (> 1 month, Socorro et al. 2016) is much longer than its estimated value in the gas phase alone (0.1 days), so this insecticide can be subjected to long-range transport and reach remote parts of the globe (Socorro et al. 2016). This observation can be extended to other insecticides since lifetimes of organic compounds in aerosol particles are much longer than in the gas phase. Residues are slowly degraded in the presence of ozone and highly reactive hydroxyl radicals. The reason is that reactions in the particle phase are limited by uptake of oxidants and their diffusion into the particle (Shiraiwa et al. 2011).

The toxicity of dust from coated seeds was evaluated for honeybees. The particulate matter was applied to plants and it showed highly toxic effects to the exposed honeybees at 0.25 and 1.0 g a.i./ha (Pistorius et al. 2015). No information is available about dust toxicity to wild pollinators, which might show different effects on different pollinator species (Rundlöf et al. 2015). Experience with exposure of honeybees to contaminated dusts has shown that wings must be evaluated as an additional contamination surface for honeybees (Poquet et al. 2015).

Finally, it is interesting to note that drilling machines using modified deflectors have low efficiency and, in the best cases, only retain a part of the dust released into the air, thus increasing the amount deposited onto the ground.

### Soil

Most of the soil contamination with neonicotinoids is expected to result from coated seeds and granular products for soil treatment, since only a fraction of the foliar sprays applied over a crop reaches the soil. A Canadian study analyzed soil samples (top 5 cm) from commercial corn fields before and immediately after planting of seeds treated with thiamethoxam or clothianidin. The mean total neonicotinoid residue before planting was 4.02 ppb (range 0.07 to 20.30 ppb), as the fields had been treated during the preceding years (Schaafsma et al. 2015). This may represent a substantial route of exposure to neonicotinoids not only for soil organisms, but also for flying insects (Bonmatin et al. 2003). Recently, Henry et al. (2015) measured imidacloprid in dietary nectar at 0.1–1.0 ppb in 13 out of the 17 surveyed honeybee colonies and at 0.1–1.6 ppb in floral nectar samples from 52 out of 82 oilseed rape fields, despite the fact that imidacloprid was not used in those fields. In the Canadian study, concentrations in soil more than doubled to 9.94 ppb (range 0.53 to 38.98 ppb) immediately after planting of the treated seeds (Schaafsma et al. 2015). The same authors estimated the persistence of residues in the fields treated according to standard agricultural practices in corn production, using application data of neonicotinoid-coated seeds over 8 years. The estimated half-life based on the history data was 0.64 years (about 8 months), longer than for two consecutive years alone (2013–2014), which was determined as 0.4 years (~ 5 months). According to the authors of that study, residues of clothianidin (the main residue in soil) might be kept stable in the fields by crop rotations between maize, soybean, and winter wheat over several years. They based their assertion in 3–4-year residue data for total neonicotinoid insecticides, which tend to plateau to a mean concentration of less than 6 ppb in agricultural soils in southwestern Ontario (Schaafsma et al. 2016). A similar finding was reported by Xu et al. (2016) who determined average clothianidin residues in soil of 7 ppb in corn fields of the Midwest USA that had been treated with coated seeds (6 ng/g seed) between 2 and 11 years, the residue levels reaching a plateau after approximately 4 years. For the treated oilseed rape seeds, the same authors reported average concentrations of 5.7 ppb clothianidin in soil from 27 Canadian fields after 2 or 4 years. Another study on corn fields in Midwest of the USA, treated with clothianidin-coated seeds at two different rates (0.25 and 0.5 mg/seed), found maximum residue levels of 11.2 ppb in soil after planting at the highest rate. Residues in soil declined and stabilized at 2 ppb after a 2-year period, with estimated half-lives of 164 and 955 days for the highest and lowest rates, respectively (de Perre et al. 2015). In Europe, a study of thiamethoxam in 18 soils found half-lives in the range 7.1 to 92.3 days (geomean 31.2 days) (Hilton et al. 2016). In this study, the rate of dissipation was not significantly affected by application type, cropped or bare soil fields, or repeated applications, or with characteristics such as soil pH and organic matter content. Soil photolysis and leaching were also negligible. Because most of the dissipation was assumed to be by microbial degradation, this may explain the presence of the metabolite clothianidin, which is more persistent than and as toxic as the thiamethoxam parent compound. Aerial sprays of dinotefuran on rice fields are still common in Japan, and this form of application typically results in residues of 25–28 ng/g (dry weight) in the paddy soils, which degrade with an average half-life of 5.4 days (Yokoyama et al. 2015).

It is known that residues of imidacloprid on surfaces can be degraded rapidly by photolysis (Wamhoff and Schneider 1999), with variable half-lives depending on the outdoor light intensities (Lu et al. 2015). The photolytic lifetime of imidacloprid at a solar zenith angle of 35° was calculated as 16 h, forming imidacloprid-urea (84%) desnitro-imidacloprid (16%) and gaseous nitrous oxide in a thermally driven process (Aregahegn et al. 2016). Although the desnitro-imidacloprid is formed in lower yields on surfaces than in aqueous solution, this metabolite can be important for mammalian toxicity (Lee Chao and Casida 1997) due to its higher binding affinity to nAChR sites. Direct soil photodegradation is only possible in the top soil surface, typically in the photic depth of soils (usually 0.2–0.4 mm). Dinotefuran and thiamethoxam exhibit a biphasic photodegradation on soil surfaces, with rate constants of 0.0198 and 0.0053 h^−1^ for the respective compounds during the first 7 h and 0.0022 and 0.0014 h^−1^ during the second phase, respectively (Kurwadkar et al. 2016).

A characteristic of some neonicotinoids is their fast degradation under anoxic soil conditions (i.e., flooded soils), which markedly contrasts with their slow dissipation in aerated soils. Mulligan et al. (2016b) have shown that the half-life of clothianidin in aerobic soils from rice fields in California exceeds 187 days (25 °C), whereas it decreases to 28.3 days (25 °C) when the same soil is flooded, reducing even further to 9.7 days under warmer conditions (35 °C). The same study found no difference in the dissipation of clothianidin from autoclaved soils or nonsterile aerobic soils, demonstrating that microbes are not a factor involved in the degradation of this neonicotinoid. This behavior is in contrast with that of thiamethoxam, which can be degraded by *Bacillus aerophilus* and *Pseudomonas putida* in soils (Rana et al. 2015). Laboratory cultures (37 °C) of these soil microbes were capable of degrading 50 mg/kg thiamethoxam in soil by 38% (*P. putida*) and 45% (*B. aerophilus*), with no production of metabolites.

Dissipation of two novel neonicotinoids, cycloxaprid and paichongding, in anoxic, flooded soils from China has been studied. All cycloxaprid enantiomers were degraded within 5 days, while its various transformation products remained in the soil up to 100 days after treatment (Liu et al. 2015). The half-life of paichongding under these conditions was estimated between 0.18 and 3.15 days (Li et al. 2016b). However, using ^14^C-cycloxaprid, more than 60% of the radioactivity in the total extractable residue was found in the water phase, suggesting that, under such experimental conditions, the initial residues of ^14^C-cycloxaprid were readily available for leaching or offsite transport (Liu et al. 2016). In a similar experiment, breakdown of four stereoisomers of paichongding was faster under acidic conditions (pH 4.1, red-clay) and slower in alkaline soils (pH 8.8, coastal saline) than in neutral loamy yellow soils. The enantiomers (5S,7R)- and (5R,7S)-paichongding were preferentially degraded in soils compared to (5R,7R)- and (5S,7S)-paichongding (Li et al. 2016a). In any case, half-lives for this compound under anaerobic, flooded soil conditions in the laboratory were rather short (< 1 to 3.7 days) (Li et al. 2016b), in agreement with the behavior of other neonicotinoids.

Unfortunately, aerobic conditions prevail in most agricultural soils and this may explain the longer persistence of neonicotinoids in this medium. For example, half-lives of imidacloprid, applied as soil drench to sandy soils from Florida and incubated in the laboratory, were estimated between 1 and 2.6 years (Leiva et al. 2015). Residues of five major neonicotinoids were determined in 52 soil samples from cocoa farms in Ghana. Some 54% of soil samples contained imidacloprid, the main neonicotinoid found, at levels between 4.3 and 251.4 ppb, 10% of the samples contained clothianidin (from 9.8 to 23.1 ppb), while the other three compounds were below the analytical limit of detection (Dankyi et al. 2014). Fipronil on seed-coated cotton (7.5 g/100 kg seed) moved into the soil and produced residues of 40 to 650 ppb, which dissipated with half-lives between 7.2 and 21.7 days (Wu et al. 2017).

Nursery-box treatment is a common application method used in rice. Boulange et al. (2016) developed a model to simulate fate and transport of fipronil and imidacloprid in rice paddy following nursery-box-applied pesticides. The hourly predicted concentrations of imidacloprid and fipronil were accurate in both paddy water and 1-cm-deep paddy soil. Levels of 2.5 μg/L for fipronil and 5 μg/L for imidacloprid were measured in paddy water and 150 ppb for fipronil and 300 ppb for imidacloprid in soil. Higher residues were found when the insecticides were applied before transplanting of seedlings rather than at sowing, but this is likely to be due to the lower amounts of insecticide applied at sowing. In China, residues of guadipyr in soils of paddy fields (up to 50 ppb) dissipated with average half-lives in the range between 0.24 and 3.33 days at three different field sites (Liu et al. 2014).

#### Sorption and leaching

In order to understand the mobility of residues from soil to water via leaching, the sorption properties of systemic insecticides need to be known. Singh et al. (2016) studied the sorption and desorption of fipronil in soils at varying concentrations, ionic strengths, temperatures, and pH values. The sorption of fipronil onto soils appeared to be a physical process with the involvement of hydrogen bonding; sorption-desorption of fipronil varied with ionic strength of the soils, while high temperatures—not pH—promoted desorption. As expected from an insecticide with a relatively high partitioning coefficient (log *K*_ow_ = 3.75), soil with higher organic content decreased the desorption rate of fipronil. Soil-water partitioning of clothianidin, determined by the batch equilibrium method in sandy and loamy soils from rice fields in California, indicates little sorption capacity of this compound and conversely its great leaching capacity. Partitioning coefficients (*K*_d_) ranged 5.1 to 10.8 L/kg, while normalized organic-carbon coefficients (log *K*_oc_) were between 2.6 and 2.8 L/kg. It was concluded that bound residues do not readily desorb, as hysteresis was observed in the four soils tested at two temperatures (22 and 37 °C).

Another method of delivery of neonicotinoids to vegetable crops is through in-furrow treatment to manage early season herbivorous pests. This type of application was used with thiamethoxam in potato crops in Wisconsin (USA) to study the movement of its residues through the soil profile during 6 months. Groundwater thiamethoxam residues increased from 0.31 μg/L early in the crop season to 0.58 μg/L after crop harvest, when its metabolite clothianidin could also be measured at 0.22 μg/L (Huseth and Groves 2014). It should be noted that residues were recycled through the groundwater irrigation system of the region. Losses by leaching were evident in corn fields of the USA, as samples of infiltration water showed levels of clothianidin in the range 10–50 ng/L (maximum 203 ng/L) throughout the seasons (de Perre et al. 2015).

The leaching ability of neonicotinoids also depends on the water solubility and persistence of the individual compounds. Leaching of neonicotinoids from sugar beet dressings was measured in the fields of Switzerland. Peak concentrations in the first precipitation event were 2830 ng/L for thiamethoxam and 1290 ng/L for imidacloprid, with levels of both insecticides declining in subsequent precipitations. Mass recoveries of neonicotinoids in the drainage water (1.2% thiamethoxam and 0.48% imidacloprid) were the highest among all pesticides found and indicate that subsurface tile drains contribute to surface water contamination with neonicotinoids from seed dressings (Wettstein et al. 2016). Available data on flupyradifurone suggest that it is persistent, and dissipation from surface soils often exceeded 1 year in field studies (90% decline). It has also the potential to reach the aquatic environment through runoff, erosion, and leaching to groundwater (US EPA Environmental Protection Agency 2014). Having a groundwater ubiquity score (GUS) index of 3.53, flupyradifurone has similar leachability to that of imidacloprid and the potential to cause analogous contamination of water resources (IUPAC database 2016).

### Water and sediments

The most common route for environmental contamination of water from systemic pesticides used in agriculture is through foliar and soil runoff into surface and/or groundwater (Bonmatin et al. 2015). Numerous surveys in recent years have shown the widespread contamination of waters with neonicotinoids, while only a few have detected fipronil. The following is an account of the recent reports on this important issue.

A nationwide Canadian study proved that the occurrence of neonicotinoids in stream discharge was correlated to precipitation patterns, with the peak concentrations of the most common insecticides (imidacloprid, clothianidin, and thiamethoxam) varying between the seasons and the type of agricultural practices. Water contamination is rife, and at two sites in Ontario, the Canadian Federal freshwater guideline value for imidacloprid (230 ng/L) was exceeded in about 75% of the samples collected (Struger et al. 2017). Schaafsma et al. (2015) analyzed 76 water samples within or around the perimeter of 18 maize fields for residues of these neonicotinoids that could have impact on bees drinking from puddles or drains. Clothianidin was found in 100% of water samples (average 2.28 μg/L and maximum at 43.6 μg/L) and thiamethoxam in 98% of the samples at average 1.12 μg/L and maximum at 16.50 μg/L. Although these average concentrations are sublethal for bees that may be exposed to the contaminated waters, they are above the safety threshold limits for aquatic organisms (Anderson et al. 2015; Morrissey et al. 2015). It was noticed that the total concentrations of these neonicotinoids in water in the agricultural fields increased 6-fold during the first 5 weeks after planting of corn, and then went down to levels similar to those before planting. In the areas surrounding the fields, residues in water were at lower concentrations than in the middle of the agricultural land and remained almost constant throughout the 2-month period of the study (Schaafsma et al. 2015). During a 3-year monitoring study in corn fields treated with clothianidin in the USA, maximum concentration of this insecticide in runoff water was found at 850 ng/L after the first storm following planting, but typical concentrations in runoff were in the range up to 200 ng/L, similar to those in the soil (de Perre et al. 2015). Runoff losses of thiamethoxam and clothianidin from seed-treated corn and soybean crops in Canada were quantified over a 2-year period. About 3% of the applied thiamethoxam was exported in runoff, with 47% of the losses found in the drains. Median concentrations of thiamethoxam were 460 and 160 ng/L (ppb) for surface runoff and drains, respectively, and for clothianidin, the corresponding concentrations were 0.02 and 10 ng/L. The highest concentrations were obtained in samples collected in the first storm postplanting: for thiamethoxam, 2200 and 440 ng/L in surface runoff and drains, respectively; for clothianidin, 70 ng/L in runoff and 50 ng/L in drains (Chrétien et al. 2017).

In Japan, aerial application of dinotefuran to paddy fields resulted in concentrations of this insecticide in paddy waters between 290 and 720 μg/L, and 10 μg/L in the adjacent river water, proving that aerial drift is still an issue. Dinotefuran half-life in paddy water was estimated close to 12 days (Yokoyama et al. 2015). In rice fields of Vietnam, imidacloprid concentrations in water ranged up to 53 μg/L, while paddy soil concentrations were up to 9 ppb. Losses of imidacloprid to the stream were in the range between 21 and 68% of applied mass. This resulted in concentrations of imidacloprid up to 83 μg/L in the streams of the watershed under the current management practices (La et al. 2015). By contrast, in Chinese rice fields, residues of guadipyr in paddy water dissipated rapidly, with estimated half-lives in the range 0.22–0.37 days (Liu et al. 2014).

Another route of transport and dispersion is snow thawing. In Canadian wetlands (Saskatchewan), neonicotinoid residues have been detected in water during early spring, after thawing of ice and before crop planting. Obviously, such residues are from previous year, suggesting they are stored in the soil beneath and removed by the melting waters (Main et al. 2016). The authors investigated the source of such residues, by studying 16 agricultural fields, selected on the basis of the previous year’s crop and the wetlands they discharged into. Neonicotinoid concentrations (clothianidin and thiamethoxam) were highest in meltwater from treated canola fields (average 267 ± 72.2 ng/L; maximum 633 ng/L), and they correlated with the spring residue concentrations found in the wetlands nearby. The bottom-layer snow of untreated fields contained residues at average 36.1 ± 9.18 ng/L, while soil particulate matter in treated canola fields showed average clothianidin residues at 10.2 ± 1.82 ppb. Persistence of neonicotinoids in colder climates are likely to contaminate wetlands even before seeding occurs through transport by snowmelt and particulate to surface water runoff during spring.

Englert et al. (2017a) studied remobilization of neonicotinoid residues from senescent foliage falling from treated trees into surface waters in Germany. They analyzed residues in foliage from black alder trees treated with one of three neonicotinoid insecticides (imidacloprid, thiacloprid, or acetamiprid) at five concentrations and developed a model to predict insecticide concentrations over a stream distance of 100 m long. They found imidacloprid water concentrations up to ∼ 250 ng/L, thus exceeding maximum permissible concentration of 8.3 ng/L for ∼ 6.5 days. Moreover, dietary uptake was identified as an additional exposure route for aquatic organisms. In addition, neonicotinoid treatments in June resulted in measurable foliar residues at the time of leaf fall (i.e., October), 4 months after application. Residue levels significantly depended on the dose and compound applied, as well as the physiological parameters of the trees. Englert et al. (2017b) reviewed the literature on foliar residues of neonicotinoids and found that they ranged between 1000 and 6000 ppb for soil and trunk application in deciduous trees and 80 and 300 ppb for soil and trunk applications of evergreen trees.

In Europe and America, the most frequently detected pesticides in surface waters (> 10% of sites) are herbicides and their metabolites. However, fungicides are even more frequent in Germany and the Netherlands, while particular insecticides were the most frequently detected compounds in certain countries: γ-HCH in France, fipronil in the USA, and imidacloprid in the Netherlands. This reflects the patterns of usage in each country (Schreiner et al. 2016). Regarding neonicotinoids, the most recent review of surveys in 11 countries found the frequency of detections between 13% (acetamiprid) and 57% (dinotefuran) of their surface waters and current residue levels ranging from an average 80 ng/L (dinotefuran) to 730 μg/L (imidacloprid). Both frequency and residue levels showed increasing trends over the past 10 years, in agreement with their increasing use as pest control products all over the word (Sánchez-Bayo et al. 2016a).

Contamination of surface water from 38 streams across the USA was assessed for 719 compounds, of which 389 were detected and quantified. Eight out of the 10 most frequently detected chemicals were pesticides, including one metabolite of fipronil (desulfinylfipronil, 0.1–10 ng/L), which was detected at 84% of the sites, whereas fipronil parent compound was found in 45% of sites at concentrations in the range 7–110 ng/L. Among the neonicotinoids detected, imidacloprid was present in 37% of sites (5–100 ng/L), clothianidin in 24% (3–70 ng/L), dinotefuran in 13% (5–110 ng/L), and acetamiprid only at one site (30 ng/L) (Bradley et al. 2017) and at least one neonicotinoid in 53% of the water samples collected from streams (Hladik et al. 2014; Hladik and Kolpin 2016). Waterborne levels of clothianidin and thiamethoxam residues were correlated to the percentage of crop land in the regions surveyed, whereas imidacloprid levels were related to the percentage of urban area within the basin (Hladik and Kolpin 2016). In five urban creeks that discharge into a brackish marsh area of San Francisco Bay, peak concentrations of insecticides were 9.9 ng/L (bifenthrin), 27.4 ng/L (fipronil), 11.9 ng/L (fipronil sulfone), 1462 ng/L (imidacloprid), and 4.0 ng/L (chlorpyrifos) (Weston et al. 2015). However, these insecticide residues enter the channels of the marsh and are diluted to the point of not showing acute toxicity to standard test species (*Hyalella azteca* and *Chironomus dilutus*).

Fipronil is found in most American surface waters and sediments due to its frequent use for urban and agricultural pest control. In the attempt to identify the main sources of contamination by this insecticide, surveys in North Carolina found that fipronil was present in almost all samples, and concentrations were substantially elevated (10–500 ng/L) near wastewater treatment plant drain pipes (McMahen et al. 2016). In Californian watersheds, residues of fipronil and its derivatives in surface water are typically in the range 2–13.8 ng/L (Sengupta et al. 2014). A survey of the Santa Clara River (California) found maximum concentrations of pyrethroids (bifenthrin and permethrin), polybrominated diphenyl ethers (PBDEs), and derivatives of fipronil in sediment (from LOQ to 6.8 ppb) that exceeded the threshold levels established for freshwater and estuarine sediments in California, which for fipronil are established as 0.09 and 6.5 ppb dry weight, respectively (Maruya et al. 2016). In a 4-year monitoring study of water quality, 60% of water samples taken from wetlands of the Prairie Pothole region of Iowa contained pesticide residues, with herbicides (chloroacetanilide and atrazine) and neonicotinoids being the most commonly found. Among the latter, clothianidin was the most frequently detected (98% samples), followed by thiamethoxam (54%) and imidacloprid (48%). Average residue levels in water were 310, 290, and 40 ng/L for the respective compounds (Evelsizer and Skopec 2016). Also in Iowa, clothianidin, imidacloprid, and thiamethoxam were ubiquitously detected in finished water samples (drinking water quality) at concentrations ranging from 0.24 to 57.3 ng/L (Klarich et al. 2017).

Another study, conducted in the Gironde estuary (France), showed the significant presence of neonicotinoids and fiproles (fipronil and its derivatives in the 0.1–8-ng/L range) in this river, even if 10 years had passed after the total ban of all agricultural uses of fipronil in France (Cruz 2015). This suggest that fipronil, used only for treatment of pets and against termites in that country, still has an impact on aquatic organisms, since its predicted no-effect concentration (PNEC) is 0.77 ng/L.

In a Brazilian reservoir that retains water from the surrounding agricultural region, 12 out of 31 pesticides and pharmaceuticals analyzed were detected; fipronil was found in 91% of the samples at average concentrations of 1.4 ng/L, and imidacloprid average residues were 2.1 ng/L in 31% of the samples (López-Doval et al. 2017). All 15 samples taken from a stream in the Mato Grosso (Brazil) that has been monitored for water quality contained residues of thiamethoxam at average concentrations of 1400 ng/L (Rocha et al. 2015). In Portugal, all 18 samples from the Arade estuary contained residues of imidacloprid up to 8 ng/L (Gonzalez-Rey et al. 2015).

In 2-year surveys of three major agricultural basins of Spain, imidacloprid was found in 17–58% of the samples from the Guadalquivir River (range 1.8–19.2 ng/L) (Masiá et al. 2013), in 37–45% of water samples from the Ebro River (range 1.1–15 ng/L) (Ccanccapa et al. 2016), and in 64–78% of the samples from the Llobregat River (range 2.1–66.5 ng/L) (Masiá et al. 2015), with the highest concentrations in all three basins corresponding to the second year of the survey. In a rural area dominated by forests and arable land in Central Germany, 19 streams were monitored for pesticide contamination using passive samples (Chemcatcher). Residues of imidacloprid and thiamethoxam in water were found in 32 and 10% of sites at concentrations in the range 9–20 and 32–44 ng/L, respectively (Münze et al. 2015).

In Vietnam, drinking water from the Mekong River Basin is contaminated with numerous pesticide residues. A survey of surface waters, groundwater, and public pumping stations showed that 98% of all 260 samples analyzed contained at least one pesticide. Fipronil was detected in 83% of the samples at average and maximum concentrations of 170 and 410 ng/L, respectively. In contrast, thiamethoxam was present only in 4% of the samples, although at higher concentrations: 630 and 950 ng/L (Chau et al. 2015).

In China, sediments from the Jiulong River in Fujian Province showed contamination with imidacloprid (87.5% samples) and acetamiprid (62.5% samples) at average concentrations of 141 and 162 ppb (dry weight), respectively (Chen et al. 2015).

### Plants and apicultural produce

The amount of neonicotinoids on treated seeds is highly variable. A study of six sunflower varieties seed-coated with thiamethoxam showed concentrations of this insecticide in the hull ranging from 17 to 39,100 ppb, which were much higher than in the kernels (range 2 to 340 ppb), as could be expected. Surprisingly, the metabolite clothianidin was also detected at similar levels: 4 to 34,700 ppb in the hulls and <LOQ to 29 ppb in the kernels (Sánchez-Hernández et al. 2016). Translocation of clothianidin from seed treatments to plant tissues was studied in corn (*Zea mays* L.) over 2 years. A maximum of 1.34% of clothianidin in the initial seed treatment was successfully recovered from plant tissues in both years and a maximum of 0.26% was recovered from root tissue (Alford and Krupke 2017). For oilseed rape seeds treated with clothianidin, plant bioavailability was 6% of clothianidin present in soil residues (Xu et al. 2016). These findings are consistent with similar resorption studies of imidacloprid in crop plants (Stamm et al. 2016; Sur and Stork 2003), thus confirming that the bulk of systemic insecticides in coated seeds remains in the soil of the field. These findings raise serious questions about the poor efficiency of the system and the inevitable environmental contamination they produce (Bonmatin et al. 2015; Goulson 2013; Sánchez-Bayo 2017).

Translocation of pesticides in plants depends on many parameters like plant morphology and physiology as well as chemical properties of the specific compounds (Bonmatin et al. 2015) and presence of adjuvants, making it a phenomenon intrinsically difficult to predict. Stamm et al. (2016) studied the uptake and translocation of imidacloprid, clothianidin, and flupyradifurone in seed-treated soybeans. They found that the novel flupyradifurone is absorbed at a higher rate during the early growth stage of the plant compared to clothianidin and imidacloprid. Conversely, there were no significant differences in compound absorptions during advanced growth states. Additionally, soil moisture stress had a positive effect just on the distribution of flupyradifurone in the leaves.

The translocation of fipronil from treated cotton seeds to young seedlings (10–15 cm height) resulted in variable residues between two consecutive years: up to 48 ppb for the first year and 646 ppb for the second year (Wu et al. 2017). Total fiprole residues in the cotton plants declined with half-lives of 2.1–7.3 days during a 3-week period and contained mostly the parent compound and low levels of three metabolites.

After spraying the rice paddies with thiamethoxam at recommended rates (30 g/ha), rice seeds collected between 5 and 35 days after flowering contained the following residues: 158 to 195 ppb (hull), 136–192 ppb (bran), and 1.2–2.2 ppb (polished rice). Neither doubling the rate of application nor applying two sprays during the season resulted in significant differences in the level of rice residues obtained (Teló et al. 2015). Residues of guadipyr in rice hulls were between 10 and 470 ppb, and they peaked up to 70 ppb in husked rice and up to 110 ppb in rice straw (Liu et al. 2014). Residues of thiamethoxam in mango fruits, after single or double foliar spray applications at recommended rates in India (0.008 and 0.016%), were measured at 1930 and 3710 ppb, respectively, 1 h after spraying. After 20 days, residues declined to 80 and 130 ppb for the single and double applications, and no residues were detected after 40 days. This suggests that preharvest withholding periods of 7 to 11 days do comply with the maximum residue limit (500 ppb) for this produce (Bhattacherjee and Dikshit 2016).

Balfour et al. (2016) measured residues of thiamethoxam and its main metabolite clothianidin in oilseed rape and maize grown from treated seeds. Neonicotinoid concentrations were found to decrease significantly with increasing plant weight. Concentrations in plant tissues roughly halved with a 4-fold increase in plant weight. In the case of hemlocks, the study from Benton et al. (2016) showed more complicated trends between residue levels and plant size depending upon dosage. When all hemlocks were given low-dose treatments, no significant differences in imidacloprid plant tissue concentrations were detected between plant size classes. However, larger hemlocks showed lower concentrations of olefin metabolite. When larger hemlocks were administered with high-dose treatments of imidacloprid, they exhibited higher imidacloprid and olefin concentrations. Metabolite concentrations were higher in high-dose compared to low-dose treatments and followed a significant linear relationship with the concentration of the parent compound in individual branchlets.

dos Santos et al. (2016) studied fipronil concentrations in the substratum and roots of *Eucalyptus* seedlings treated in the nursery box. They found that irrigation up to 56 days, performed in the nurseries, did not decrease the fipronil concentration in the seedlings; thus, nursery-box treatment may reduce costs and environmental dispersion of the active ingredient.

Turfgrass with white clover was directly sprayed into the bloom using two neonicotinoids for the control of grubs (Larson et al. 2015). Nectar from directly sprayed clover blooms contained 5493 to 6588 ppb imidacloprid or 2882 to 2992 ppb clothianidin. Imidacloprid residues in turfgrass guttation averaged 88 ppb at 1 week after treatment, a level still toxic to natural predators such as *Orius insidiosus*, and declined to 23 ppb within 3 weeks. Mowing of the blooms decreased the residue loads by 99.4% (imidacloprid) and 99.8% (clothianidin), thus reaching levels below the acute toxicity to the predatory insects (Larson et al. 2015).

Guttation fluid from winter oilseed rape plants grown from seeds coated with neonicotinoids (clothianidin 10 g/kg seed; thiamethoxam 3.6–4.2 g/kg seed) contained residues of the insecticides up to 130 μg/L in autumn and <LOD to 30 μg/L in winter. In the following spring, residues of clothianidin in guttation droplets were similar to those during winter, declining to <LOD until the time of flowering. These levels are lower than those reported by the same authors for clothianidin in guttation fluid of seed-coated maize, which were up to 8000 μg/L (Reetz et al. 2011).

For pollen/beebread and nectar/honey, an exhaustive list of published data was given in Bonmatin et al. (2015) where averaged values, measured worldwide for neonicotinoids and fiproles, have been reported. For instance, averaged imidacloprid residues in treated fields were up to 39 ppb (pollen/beebread) and 73 ppb (nectar/honey) in the available literature, contrasting with the average maximum residue values of 6.1 ppb for pollen and 1.9 ppb for nectar reported by Godfray et al. (2015). A recent report (IPBES 2016a) has confirmed that exposure to nectar and pollen residues is highly dependent on agricultural practices and specifically on the mode of application of neonicotinoids (seed-dressing, foliar spray, soils drenches, etc.); other factors include the application rate, crop, variety, or location (IPBES 2016b). Thus, while Rundlöf et al. (2015) reported average residues of 10.3 and 13.9 ppb clothianidin in nectar and pollen from oilseed rape crops, Rolke et al. (2016) found only 0.72 and 0.73 ppb in the same matrices from the same type of crop. Interestingly, the IPBES report mentioned that residues can be 10–20-fold greater when the same neonicotinoids are applied as foliar sprays at a similar rate per hectare or as soil drenches. High neonicotinoid residues have also been measured in other crops treated differently, and even for not treated plants (Botias et al. 2015). A summary of residues is shown in Table 3.

**Table 3:** Residues of neonicotinoids and fipronil in agricultural products and animal samples. Values (in ng/g) indicate average and maximum (in brackets) residues and their frequency of detection (%), unless a range is specified. The data on pollen/beebread and nectar/honey complement the exhaustive review of residues previously published (Bonmatin et al. 2015)

	Acetamiprid	Clothianidin	Guadipyr	Imidacloprid	Nitenpyram	Thiacloprid	Thiamethoxam	Fipronil	References
Products									
Beebread		7.2 (18.4)							Pistorius et al. (2015)
Beebread		12		0.5 (1.5)		0.2 (1.8) 29.3%	1.7		Parrilla Vázquez et al. (2015)
Beebread	(171.4) 30%	< 5%		25%		(177) 96%	25%		Daniele et al. (2017)
Beebread		5.2 (15.7) 58%		4.2, 5%	4.5 (10.5) 26%	< 0.1	28.7 (62.5) 21%		Codling et al. (2016)
Beeswax				< 1, 2.5%		(3.4) 26%	(106.5) 3%		Daniele et al. (2017)
Beeswax	1–4, 6%			3.0–5.1, 5%		4.0–10.4, 3%		1.0, 1%	López et al. (2016)
Honey		13.7 (192.8)							Gbylik-Sikorska et al. (2015)
Honey		0.25 (0.82) 72%		<0.1			0.27 (0.79) 68%		Jones and Turnbull (2016)
Honey		6.7 (20) 68%		1.1 (6.2) 32%	< 0.1	14.4, 4%	19.4 (41.1) 75%		Codling et al. (2016)
Honey		1.35							Rolke et al. (2016)
Cabbage		74 (724)							Li et al. (2014)
Honeysuckle leaves						22 (4400)	17 (3200)		Fang et al. (2017)
Mango fruit				80 (3710)					Bhattacherjee and Dikshit (2016)
Oilseed rape plants		<LOD–6.5							Rundlöf et al. (2015)
Rice grain (bran)			20 (101)				131 (244)		Teló et al. (2015), Liu et al. (2014)
Rice grain (hull)			80 (470)				143 (225)		Teló et al. (2015), Liu et al. (2014)
Rice grain (polished)							1.2 (4.0)		Teló et al. (2015)
Winter melon				10 (210)					Huang et al. (2015)
Animals									
Amphipods						0.1 (0.39)			Inostroza et al. (2016)
Bumblebees	< 0.01–0.17, 0.7%	< 0.48–1.4, 0.7%		< 0.7–10, 7%		< 0.02–1.17, 2%	< 0.3–2.3, 6%		Botías et al. (2017)
Eels								4.0–20*	Michel et al. (2016)
Honeybees	(10) 5%	2.5%		(1.7) 9%		(1.6) 13%	8%		Daniele et al. (2017)
Honeybees		6.5–33							Pistorius et al. (2015)
Honeybees	1.7–8.2	5.3–76.2		3.3–174		21.9–28.8	588	232–590	Kiljanek et al. (2016)
Honeybees		2.5–7.1		0.1–11.1*					Codling et al. (2016)
Honeybees		4–13.1		4.5–27*					Gbylik-Sikorska et al. (2015)
Honeybees							0.3–0.95		Reetz et al. (2016)

*Total residues of parent compound and metabolites

New and sophisticated analytical methods have been developed to detect pesticide residues in hive matrices (pollen, nectar, beebread, wax) and even at sub-ppb levels in bees or in individual bumblebees (David et al. 2015; Valverde et al. 2016). Multiresidue analysis of 41 pollen samples collected from apiaries in agricultural areas of Spain between 2012 and 2015 showed residues of at least 2 pesticides and a mean of 6 pesticides per sample in the range 3.7–1856 ppb (Parrilla Vázquez et al. 2015). The most commonly found chemicals were used for control of *Varroa destructor* (coumaphos, tau-fluvalinate, chlorfenvinphos, in 44–73% samples), followed by two fungicides (carbendazim and thiabendazole), the organophosphorus chlorpyrifos, and the neonicotinoid thiacloprid (29.3% samples). Note that many samples were taken during the moratorium on imidacloprid, thiamethoxam, and clothianidin in Europe. In a different study, pollen collected from bean plants, strawberry, and raspberry fields contained neonicotinoids up to 67 ppb and fungicides up to 14 ppb, although the detection frequency per sample was higher for the fungicides (David et al. 2015). The same authors measured concentrations of these pesticides in pollen of oilseed rape and wildflowers grown near arable fields and compared them with residues in pollen collected by honeybees and bumblebees in agricultural and urban settings (David et al. 2016). Oilseed rape pollen contained high concentrations of most pesticides (median 3.8–7.5 ppb neonicotinoids and 2.5–58 ppb fungicides). Surrounding wildflowers were frequently contaminated though at lower levels: average 0.13–0.5 ppb neonicotinoids and 0.1–8.5 ppb fungicides. Various pollen collected by honeybees during the oilseed rape bloom contained averaged neonicotinoid residues in the range 0.15–0.9 ppb and those of fungicides in the range 0.3–12.0 ppb. In a follow-up study, levels of neonicotinoid residues in foliage of oilseed rape plants treated from coated seeds were measured as 1.4–11 ppb, while the levels in pollen from the same plants ranged 1.4–22 ppb (Botías et al. 2016). However, these authors found that the vast majority (97%) of neonicotinoids brought back in pollen to honeybee hives in arable landscapes came from wildflowers, not crops (Botias et al. 2015). A similar finding was reported in North American agricultural regions, where pollen from corn and soybeans represented 17.6 and 6.3% of the total amount collected by honeybees and, thus, constituted only a tiny fraction of the diversity of pollen resources used by the bees (Long and Krupke 2016). In some cases, the neonicotinoid levels in the flowers overlapped with LC_50_s reported for some beneficial insects such as polyphagous hymenopteran parasitoids and butterflies (Botías et al. 2016). Average concentrations of clothianidin in corn pollen were low (1.8 ppb) and did not appear to correlate with the total years of use or soil concentrations. The same was found with oilseed rape, for which average clothianidin concentrations in nectar were 0.6 ppb and not correlated to use history or soil concentrations (Xu et al. 2016).

A recent study performed a survey of 10 pesticide residues (including imidacloprid, thiamethoxam, acetamiprid, thiacloprid, and clothianidin) and three neonicotinoid metabolites (6-chloronicotinic acid, 5-OH, and olefinic derivatives) across France during springs 2012–2016 (Daniele et al. 2017). Three relevant bee matrices (honeybee, beebread, and wax) were investigated. In total, 488 samples were analyzed, primarily taken from symptomatic colonies. Neonicotinoids (especially thiacloprid) and boscalid were the pesticides detected the most, whatever the matrix. Wax matrix contained the highest concentrations (up to 302.3 ppb for boscalid and 106.5 ppb for thiamethoxam), whereas beebread was the matrix contaminated most frequently (77% of positive samples). Interesting comparisons between results preceding and during the partial EU moratorium have been made. In 2013, restrictions of the uses for imidacloprid, thiamethoxam, and clothianidin, as seed coating for some bee attractive plants and cereals, have been imposed by the EU Commission. Comparisons showed a significant reduction on the frequency of detection of clothianidin in honeybees, thiamethoxam in honeybees and beebread, and imidacloprid in beebread and wax. The major reductions in frequency concerned imidacloprid and thiamethoxam at low levels (< 1 ppb) in beebread. On the contrary, thiamethoxam was only observed in wax after 2013, with two of the four samples in the 1–5-ppb range, and the other two samples above 50 ppb (Daniele et al. 2017). As beeswax is commonly recycled for making the frames of commercial hives, this might explain the unexpected contamination of wax after the EU moratorium of 2013 came into effect.

Recently, also ornamental plants from garden nurseries have been analyzed for insecticide and fungicide residues and their consequent exposure risk for pollinators (Lentola et al. 2017). Leaves, pollen, and nectar from 29 “bee-friendly” plants were analyzed and neonicotinoids were detected in more than 70% of the plants. Chlorpyrifos and pyrethroid insecticides were found in 10 and 7% of plants, respectively; boscalid, spiroxamine, and DMI-fungicides were detected in 40% of the plants. In pollen samples, systemic compounds were detected at similar concentrations than in leaves: thiamethoxam, clothianidin, imidacloprid, and chlorpyrifos were present in pollen at concentrations between 6.9 and 81 ng/g, levels that overlap with those known to cause harm to bees.

### Food and beverages

Fang et al. (2017) studied thiamethoxam and thiacloprid residues in tea (*Lonicera japonica*) leaves. Half-lives of thiamethoxam and thiacloprid were 1.0–4.1 days in the honeysuckle flowers and leaves, with degradation rate constants *k* ranging from − 0.169 to − 0.696. Following application of 28–102 g (a.i.)/h m^2^, residues were 110–1370 ppb on the 7th day after treatment and between < 0.01 and 46 ppb on the 14th day after treatment on average. They studied the effect of planting, drying, and tea brewing on the residue levels. The sun- and oven-drying (70 °C) digestions were 59.4–81.0% for the residues, which were higher than the shade- and oven-drying percentages at lower temperatures (30, 40, 50, and 60 °C, which ranged from 37.7 to 57.0%). The authors concluded that after the 7th day, residue levels are low enough to be considered safe for human consumption according to regulations. However, the study did not investigate metabolites.

Huang et al. (2015) studied residues of imidacloprid in winter melon (*Benincasa hispida* var. *chieh-qua*). They found that half-lives of imidacloprid under field conditions were 3.3 and 3.5 days in Guangzhou and Nanning at a dose of 180 g (a.i.)/ha. The terminal residues of imidacloprid were from 10 to 210 ppb, which could be considered safe to human health. Also, in this study, metabolites/degradation products were not measured.

Honey is crucial for bees but is also food for humans. Analysis of residues from Polish apiaries found between 13.7 and 192.8 ppb clothianidin (Gbylik-Sikorska et al. 2015). Honey samples collected in early spring 2013 from apiaries in the vicinity of oilseed rape fields in the UK were not burdened with residues of either imidacloprid or its metabolites but contained minor clothianidin residues from < 0.02 to 0.82 ppb and thiamethoxam residues between < 0.01 and 0.79 ppb (Jones and Turnbull 2016). Finally, in honey samples collected from hives in Saskatchewan (Canada), the most frequently detected neonicotinoids were clothianidin (68%), thiamethoxam (75%), and imidacloprid (32%) at mean concentrations of 8.2, 17.2, and 1 ppb (wet mass), respectively. All pollen samples contained residues below the acute lethal risk for bees, the calculation being based on the respective acute LD_50_ for each compound (Codling et al. 2016).

### Animals

In order to determine the effects of a field application of dusts from maize seeds treated with Poncho formulation on honeybees (*Apis mellifera* L.), dust was applied at rates of 600 g/ha, corresponding to 0.25–1.0 g/ha of the a.i. clothianidin. Levels of 4.3- to 17-fold mortality compared to preapplication levels were observed, increasing during a 7-day period. Residues detected in dead bees were highest in the first 24 h of exposure (3 ng/bee), declining to about 0.5 ng/bee after a week, while median residues in beebread were similar (7.7 ppb) under both rates of exposure (Pistorius et al. 2015). In Poland, residues of 57 pesticides were quantified in dead honeybees collected from hives showing acute intoxication (Kiljanek et al. 2016) but only 48 compounds in living bees (Kiljanek et al. 2017). The pesticides most commonly found in poisoned bees were chlorpyrifos (12%), dimethoate (10%), and clothianidin (7.4%). All five neonicotinoids used in Poland and fipronil were present in dead bees at concentrations in the range 1.7–76.0 and 232–590 ppb, respectively. The only residues of these systemic insecticides reported for living honeybees were those of acetamiprid (1.2–5.4 ppb, 4.1%) and thiacloprid (1.3–14.0 ppb, 4.7%), probably because the latter neonicotinoids are less toxic to bees than imidacloprid, thiamethoxam, clothianidin, or fipronil (Sánchez-Bayo and Goka 2014).

In Saskatchewan (Canada), more than 50% of the honeybees sampled showed detectable residues of clothianidin (0.1–7.1 ng/bee), and 7% of samples had residues above the LD_50_. Imidacloprid was not detected in bees, but its metabolites were found at concentrations ranging 0.1–11.1 ng/bee, suggesting that exposure to this insecticide is greater than originally assumed (Codling et al. 2016). Rapid transformation of imidacloprid in honeybees is well known (Suchail et al. 2004); thus, the parent compound is usually hard to detect unless as a result of acute poisoning or by immediate sampling after exposure. As the poisoned honeybees from Poland showed imidacloprid residues of 27 ppb in the dead bodies, together with imidacloprid-urea at 45 ppb, the causal relationship can be identified (Gbylik-Sikorska et al. 2015).

In individual bumblebees (approx. 170 mg/bee), the method developed by David et al. (2015) was able to detect only thiamethoxam, thiacloprid, and five fungicides. In another study, five species of bumblebees (*Bombus* spp.) collected from agricultural and urban areas of Sussex (UK) showed a large array of pesticide residues, including five neonicotinoid insecticides, 13 fungicides, and one pesticide synergist. In total, 61% of the 150 individuals tested revealed detectable levels of at least one of the compounds, with the fungicide boscalid being the most frequently detected (35%). Concentrations and detection frequencies of neonicotinoids were the highest in bees collected from urban sites during early summer, contrary to the pattern found with the other pesticides. Imidacloprid (7% samples) and thiamethoxam (6%) were present in bees at concentrations ranging 0.7–10 and 0.3–2.3 ppb, respectively. Residues of three other neonicotinoids were less frequent and ranged up to 1.4 ppb (Botías et al. 2017). Honeybees ingesting guttation fluid from oilseed rape treated with thiamethoxam showed concentrations of this insecticide in their honey sacs ranging from 300 to 950 ng/L, while the corresponding concentrations in guttation fluid varied between 3.6 and 12.9 μg/L thiamethoxam (Reetz et al. 2016). Such difference in concentration could indicate that either most of the insecticide (92%) in the droplets was adsorbed by the forager bees or it had been diluted by ingestion of additional uncontaminated water.

In the eastern stretches of the Danube River in Germany, 47% of the 19 amphipods (*Dikerogammarus* spp.) collected showed residues of thiacloprid at levels 0.1–0.39 ppb (wet body weight) (Inostroza et al. 2016). Eels in the Elbe River (Germany) are exposed to sublethal fipronil concentrations in water (range 0.1–1.6 ng/L) throughout the whole year. Residues of fipronil sulfone were found in the eel liver and muscle at average concentrations of 20 and 4 ppb, respectively (Michel et al. 2016).

### Summary of findings

New research into abraded dust particles loaded with systemic neonicotinoids has revealed that they are an important source of soil contamination in the treated fields. The particles also reach the vegetation at the field margins and pose a risk to nontarget pollinators and other organisms due to their high concentrations of active ingredient.

The fate of soil residues has been studied in more detail, particularly with thiamethoxam and clothianidin used in coated seeds. Their persistence throughout the crop season is now evident, and their translocation to pollen and nectar has been measured in several studies. Dissipation of imidacloprid, dinotefuran, and the new neonicotinoids such as cycloxaprid and paichongding has also been studied in rice paddies. While photolytic degradation is important, leaching of soil residues through water infiltration is a major problem for groundwater contamination.

Water surveys in many countries have shown the widespread contamination with neonicotinoids and fipronil of agricultural drains, rural and urban streams, drinking water, and effluents from water treatment plants. Residue levels, in the parts per billion range, are increasing, as the use of these insecticides continues to grow all over the world and residues in soils and leaves from treated trees are being released into water systems.

New research on the contamination of pollen and nectar with systemic insecticides has shown the variability of residue levels between crops and wildflowers at the crop margins, and the risks the latter pose for pollinators. In contrast, little is known about residues of systemic insecticides in agricultural products, although some data on fruit, tea, and honey have been obtained in the parts per billion-parts per million range.

## Remediation

### Soil

Soil amendments with organic vermicompost have demonstrated to reduce the residence time of imidacloprid in agricultural soils of southeastern Spain. The soil half-life after incubation with an added olive-vermicompost for 3 months was 67 days, but the time for 90% reduction in soil residues was 265 days, compared to > 512 days for soils without amendment (Castillo Diaz et al. 2017). Vela et al. (2017) tested solarization and biosolarization for detoxification of soils containing acetamiprid, imidacloprid, thiamethoxam, chlorantraniliprole, and flubendiamide. The warm soil temperature after adding organic matter from sheep manure, meat-processing waste, and sugar beet vinasse increased insecticide disappearance rates compared with nontreated soils.

### Water

Photolysis of neonicotinoids is rapid in clear aqueous environments. Half-lives for dinotefuran, imidacloprid, and thiamethoxam estimated under laboratory conditions in pure water are 3.6, 2.3, and 3.8 h, respectively (Kurwadkar et al. 2016). Nitenpyram degrades in a similar way in drinking water as imidacloprid, with oxidation to olefin and other metabolites (Noestheden et al. 2016). Half-lives for thiamethoxam in a laboratory photoreactor were 0.2–1.5 days for different seasons and 0.8 days for outdoor photolysis in Manitoba, Canada (50° N latitude) (Lu et al. 2015). However, photolysis tends to be faster in pure, deionized water than in turbid environmental waters. For example, the photodegradation of clothianidin was calculated as 14.7 days in deionized water, 16.6 days in river water, and 18.0 days in water from flooded rice paddies (Mulligan et al. 2016a). Also, thiamethoxam photolysis at soil depths greater than 8 cm was negligible (Lu et al. 2015), indicating that turbidity and light attenuation are important factors controlling the photodegradation of this and other neonicotinoids under field conditions.

Conventional wastewater treatment plants (WWTPs) are quite inefficient in removing neonicotinoids from contaminated waters. For example, the concentrations of imidacloprid (60.5 ± 40.0 ng/L), acetamiprid (2.9 ± 1.9 ng/L), and clothianidin (149.7 ± 289.5 ng/L) in the influent of a WWTP in the USA were reduced by 3.3, 20.7, and 53.1%, respectively, in the effluent (Sadaria et al. 2016). In another study, fipronil and its derivatives were found in the WWTP influent at 1–88 ng/L, 62% in the water phase, with the remainder being bound to filter-removable particulates. Total fiproles persisted during the treatment, with 65 ± 11% remaining in water and the balance partitioning into sludge, with fipronil at 3.7–151 ppb dry weight (Sadaria et al. 2017). The authors identified imidacloprid, acetamiprid, clothianidin, and fiproles as recalcitrant sewage constituents that persist through WWTPs. An extrapolation of data from 13 WWTPs in the USA showed annual discharges of 1000–3400 kg of imidacloprid in treated effluents nationwide. Concerning fipronil, Gomes Júnior et al. (2017) tested heterogeneous photocatalysis on TiO_2_ nanoparticles for wastewater treatment. This method successfully degraded fipronil into four main degradation products (fipronil sulfone, fipronil sulfide, and two new products reported in Table 1) under either artificial or natural irradiation. The four degradation products have lower toxicity toward *Vibrio fischeri* compared with the parent compound. In addition, the ozonation process achieves good oxidation of thiamethoxam in water, but in order to reach 70% removal within 90 min, the ozone concentrations must range between 10 and 22.5 mg/L and the pH be in the range 5 to 11 (Zhao et al. 2016). Ozonation has been tested also for abatement of acetamiprid, which was converted into four transformation products (N-desmethyl derivative, 6-chloronicotinic acid, N′cyano-N-methyl acetamidine, and N′-cyano acetamidine). Toxicity, evaluated with a microtox bioassay, showed an increase during the ozonation process, followed by a decrease to relatively low values (Cruz-Alcalde et al. 2017). The Iowa City treatment facility used granular activated carbon filtration to remove most of imidacloprid, clothianidin, and thiamethoxam and produce finished water of drinking quality, whereas the conventional water treatment removed only about 50% of thiamethoxam and none of the other two neonicotinoids (Klarich et al. 2017).

A successful approach to mitigate the impacts of insecticide residues in water is to maintain uncontaminated stream reaches that can foster recovery of the impacted populations downstream. In Central Germany, streams contaminated with neonicotinoids and other insecticides were thus monitored for impacts on and recovery of the macroinvertebrates’ biodiversity and abundance. Forested headwaters were associated with the absence of long-term effects on the macroinvertebrate community composition, even if the most vulnerable species could still be affected at concentrations 3 or 4 orders of magnitude below the LC_50_ value for standard test organisms (Orlinskiy et al. 2015).

In an extensive survey of neonicotinoids in 238 wetlands of Saskatchewan (Canada), their detection was best explained by shallow marsh plant species identity (34.8%) and surrounding crop (13.9%), whereas concentrations of these insecticides were associated with shallow marsh plant species identity (14.9%) and wetland depth (14.2%). Thus, plant communities appear to be key drivers of neonicotinoid presence and concentration in Prairie wetlands (Main et al. 2015). Based on these findings, the authors recommend the use of buffer zones consisting of diverse native vegetation for retaining and/or minimizing neonicotinoid transport to the aquatic ecosystems. A follow-up study by the same authors investigated whether macrophyte species were capable of reducing the movement of neonicotinoids from cultivated fields into surface waters. Indeed, nonvegetated wetlands had higher detection frequency and water concentrations of clothianidin and thiamethoxam than vegetated wetlands. Neonicotinoids were detected in 43% of wetland plants and quantified in 8% of all plant tissues sampled (Main et al. 2017). The plant species with the highest absorption of residues were *Equisetum arvense* (78% plants with clothianidin, up to 2.01 ppb), *Alisma triviale* (65% plants with imidacloprid, up to 2.51 ppb), and *Typha latifolia* (45% plants with imidacloprid up to 2.61 ppb and thiamethoxam up to 8.44 ppb). The results are promising for developing mitigation strategies that may decrease neonicotinoid residue loads in wetlands.

Grass strips planted within crops can mitigate the amount of pesticide residues moving into the aquatic systems. Data collected from soybean fields planted with neonicotinoid-coated seeds in the USA have demonstrated that groundwater levels of imidacloprid, clothianidin, and thiamethoxam in crops that had grassy strips (average 11 ng/L) showed significantly lower concentrations than crops without strips (average 20 ng/L). The same was true for the soil residues, which were much lower in the fields with grassy strips (< 1 ppb) than in those without (average 6 ppb). However, the residue levels of these insecticides in surface runoff waters were variable (range 44–140 ng/L) and not statistically different among the field with or without strips (Hladik et al. 2017). Another study, using engineered wetlands constructed for removal of waterborne neonicotinoid residues, found that imidacloprid and acetamiprid could not be removed (Sadaria et al. 2016).

Bioswales can be effectively used to reduce concentrations of suspended sediments, metals, and hydrocarbons from urban runoff (Ulrich et al. 2015, 2017). In California, bioswales significantly decreased the amount of pyrethroid pesticides (74% reduction) but not fipronil, indicating that the latter insecticide may require a different removal method. Thus, the resulting treated runoff was still toxic to amphipods (*Hyalella azteca*) and midges (*Chironomus dilutus*), but not to waterfleas (*Ceriodaphnia dubia*) or fish (*Pimephales promelas*) (Anderson et al. 2016). However, in recycled wastewater samples from North Carolina treated with NaOCl for disinfection, fipronil and all its known derivatives disappeared, apparently by oxidation (McMahen et al. 2016), raising hopes for the remediation of this recalcitrant and ubiquitous insecticide.

### Summary of findings

Soil amendment with vermicompost proved useful to accelerate the degradation of imidacloprid compared with untreated soils; however, 90% reduction still required 265 days. Biosolarization for detoxification of soils also showed potential for remediation of neonicotinoids in situ.

State-of-the-art WWTP proved inefficient for removal of neonicotinoids, fipronil, and their metabolites. Conversely, tests on the use of TiO_2_ nanoparticles as catalysts for photodegradation and treatment with NaOCl showed potential for fipronil removal, while granular activated carbon filtration removed most of imidacloprid, clothianidin, and thiamethoxam to produce finished water of drinking quality.

A successful approach to mitigate the impacts of systemic insecticide residues in water is to maintain uncontaminated stream reaches that can foster recovery of the impacted populations downstream. Buffers consisting of diverse native vegetation and grass strips planted within crops can mitigate the amount of pesticide residues moving into the aquatic systems.

## Conclusions, knowledge gaps, and recommendations

Numerous research efforts have been undertaken in response to large gaps of knowledge as attested by an exponential increase in publications on neonicotinoids and bees since 2010. After publication of the Worldwide Integrated Assessment special issue (Bijleveld van Lexmond et al. 2015; Simon-Delso et al. 2015; Bonmatin et al. 2015; Pisa et al. 2015; Gibbons et al. 2015; Chagnon et al. 2015; Furlan and Kreutzweiser 2015; van der Sluijs et al. 2015), most of the additional knowledge reported in the present paper concerned (i) the mode of action and metabolism of new neonicotinoids; (ii) the synergistic effects of neonicotinoids/fipronil with other insecticides, fungicides, herbicides, and adjuvants; (iii) their interaction with honeybee viruses vectored by the *Varroa destructor* mite and the *Nosema ceranae* microsporidian parasite; (iv) the contamination of all environmental compartments (dust, soil, water, sediments, and plants) and also of bees, apicultural products, food and beverages, and animals; and (v) remediation of neonicotinoids and fipronil, especially in water.

Some publications have been criticized because of weak protocol and/or conclusions clearly opposite to the conclusions from a large set of other publications (e.g., see Hoppe et al. (2015) and Sánchez-Bayo et al. (2017)). Obviously, this raises also the issue of conflicts of interests because of the potential of large economic consequences.

As manufacturers continuously propose new pesticides for authorization and marketing, the neonicotinoid group has grown. A rationale for the classification of pesticides into chemical groups is not available, so some of the new molecules are presented for commercial reasons as pioneer compounds of a new group despite having molecular structures and modes of action analogous to already existing pesticides. For this reason, sulfoxaflor and flupyradifurone should be considered neonicotinoids despite manufacturers’ claims to the contrary.

New research concerning the mode of action of neonicotinoids revealed more complex interactions with the receptor and secondary targets in both invertebrates and vertebrates, like in the case of imidacloprid which interacts also with the GABA receptor. New metabolites and degradation products have been discovered, but a continuous research into transformation products is still required to evaluate their toxicity to nontarget organisms. This is particularly important for research into remediation, as knowledge of transformation products may trigger specific research into abatement strategies for recalcitrant by-products.

Organisms in the environment are exposed to cocktails of pesticides and other stressors. Recent research revealed synergistic interactions between pesticides, their formulations and in particular the combinations of azole fungicides and neonicotinoids/fipronil currently used in seed coatings. This exacerbates the already declining health of managed bees due to immune suppression, which promotes parasites, viral infections, and their proliferation.

In regard to the environmental contamination, the new literature since 2015 confirmed that dust produced from abraded coated-seeds during sowing remains an issue for environmental contamination by systemic pesticides and their highly toxic effects to nontarget species. Equally, the persistence of neonicotinoids, fipronil, and derivatives in soil and sediments is of serious concern, since these compartments act as a reservoir of residues that are later discharged into water.

Recent water surveys in more than a dozen countries bring to the fore the widespread contamination of surface waters around the world, with obvious impacts of neonicotinoids and fipronil on a large range of aquatic invertebrate communities (see part 2 of this review: Pisa et al. 2017).

It seems that the only way to mitigate water contamination is by the use of wetland plants or trees that may absorb the residues (Beketov and Liess 2008; Orlinskiy et al. 2015). However, such wetland plants or trees could themselves expose nontarget species to these systemic pesticides. Seriously alarming is the fact that such waterborne residues pass through the conventional water treatment facilities almost unaltered, even in developed countries with state-of-the-art cleaning technology. In particular, the residues and toxic metabolites of fipronil are recalcitrant to further degradation. Given the extent of the environmental contamination and the adverse effects on invertebrate and vertebrate communities, research is needed into new methods of abatement and environmental remediation. Further studies need to pay careful attention to the toxic transformation products.
